# GC–IMS facilitates identification of carbapenem-resistant *Klebsiella pneumoniae* in simulated blood cultures

**DOI:** 10.1186/s13568-024-01708-1

**Published:** 2024-04-24

**Authors:** Fuxing Li, Yunwei Zheng, Chuwen Zhao, Junqi Zhu, Yaping Hang, Youling Fang, Longhua Hu

**Affiliations:** 1 The Second Affiliated Hospital, Jiangxi Medical College, Nanchang University, Nanchang, Jiangxi China; 2 Department of Jiangxi Provincial Key Laboratory of Medicine, Clinical Laboratory of the Second Affiliated Hospital of Nanchang University, Mingde Road No.1, Nanchang, 330006 Jiangxi People’s Republic of China; 3 School of Public Health, Nanchang University, Nanchang, Jiangxi China

**Keywords:** Carbapenem-resistant *Klebsiella pneumoniae* (CRKP), Gas chromatography–ion mobility spectrometry (GC–IMS), Volatile organic compounds (VOCs), Imipenem (IPM), Carbapenemase (CBPM)

## Abstract

This study aimed to identify carbapenem-resistant *Klebsiella pneumoniae* (CRKP) based on changes in levels of its volatile organic compounds (VOCs) in simulated blood cultures (BCs) using the gas chromatography–ion mobility spectrometry (GC–IMS) technique. A comprehensive analysis of volatile metabolites produced by *Klebsiella pneumoniae* (*K*. *pneumoniae*) in BC bottles was conducted using GC–IMS. Subsequently, the released VOCs were analyzed to examine differences in VOC release between CRKP and carbapenem-susceptible *Klebsiella pneumoniae* (CSKP). A total of 54 VOCs were detected, of which 18 (6 VOCs found in both monomer and dimer forms) were successfully identified. The VOCs produced by *K*. *pneumoniae* in BC bottles (BacT/ALERT® SA) were primarily composed of organic acids, alcohols, esters, and ketones. The content of certain VOCs was significantly different between CRKP and CSKP after the addition of imipenem (IPM). Moreover, the inclusion of carbapenemase inhibitors facilitated the identification of carbapenemase-producing *K*. *pneumoniae* based on the variations in VOCs. This study demonstrates the utility of GC–IMS technology in identifying CRKP, and reveals that changes in VOCs are closely related to the growth and metabolism of *K. pneumoniae*, indicating that they can be leveraged to promote early identification of CRKP bacteremia. However, further in-depth studies and experiments are needed to validate our findings.

**Supplementary Information:**

The online version contains supplementary material available at 10.1186/s13568-024-01708-1.

## Introduction

Bloodstream infection (BSI) is a systemic infectious disease that threatens human life and health. It has been shown to induce bacteremia, septicemia and sepsis. In some cases, it causes shock, disseminated intravascular coagulation (DIC), multiple organ failure, and even death (Kern and Rieg 2020; Tabah et al. 2023; Timsit et al. 2020). Epidemiologic data in China have demonstrated that Gram-negative bacteria, such as *Escherichia coli* and *Klebsiella pneumoniae* (*K. pneumoniae*), are the most commonly isolated pathogens associated with BSI, accounting for over 50% of BSI diseases (Chen et al. 2023). The widespread use of antibiotics and the prevalence of carbapenem-resistant *Enterobacteriaceae* (CRE) have made it difficult to treat these infections (Fang et al. 2023; Liu et al. 2021). Carbapenem-resistant *Klebsiella pneumoniae* (CRKP) is the most prevalent CRE associated with BSI. It is prevalent in developed and developing countries, with high drug resistance and mortality rates (Fang et al. 2023; Liu et al. 2021). Notably, CRKP accounts for 60–90% of all CRE isolates in China (Hu et al. 2022). In 2021, an annual report of the Blood Bacterial Resistance Investigation Collaborative System (BRICS) indicated that the isolation rate of CRKP was 15.8% (328/2076) (Chen et al. 2023). Generally, the management of CRKP BSI is challenging because of emergence of rapid spread of multidrug resistance strains, high mortality rate, and lack of antimicrobial agents. Therefore, it is important to identify CRKP BSIs and develop appropriate antimicrobial agents to improve patient treatment.

Currently, CRKP is primarily identified using conventional antimicrobial drug susceptibility testing methods. Although new methods, such as the widely employed carbapenem inactivation method (CIM), have increased the accuracy of detecting specific CRKP carbapenemase (CBPM) type, these supplementary tests are time-consuming and often necessitate overnight culture, which delays the initiation of clinical treatments (Luo et al. 2023; Yu et al. 2022). Therefore, investigating novel approaches for the rapid detection of CRKP is imperative.

Volatile organic compounds (VOCs) are a diverse collection of low-molecular-weight, low-boiling-point, high-vapor-pressure metabolites generated by bacteria (Kai et al. 2009). Accumulating evidence indicates that VOCs are potential biomarkers for bacterial identification (Chen et al. 2017b; Drees et al. 2019; Lu et al. 2022). Research conducted by our team on bacterial metabolomics has yielded significant findings with regard to the application of rapid mass spectrometry-based detection of bacteremia pathogens based on microbial VOC fingerprints (Chingin et al. 2015, 2016; Hang et al. 2017; Hu et al. 2016; Zhong et al. 2019).

To date, most studies have primarily investigated the involvement of VOCs in pathogenic bacteria identification. Only a handful of studies have identified strains by assessing alterations in VOCs based on the drug susceptibility and antibiotic resistance mechanisms of these pathogenic bacteria. In our previous study, we achieved early identification of CBPM-producing CRKP by examining changes in 3-methyl-1-butanol levels in trypticase soy broth (TSB) cultures (Luo et al. 2023). Furthermore, another study reported that carbapenem-susceptible *Klebsiella pneumoniae* (CSKP) can be differentiated from CRKP based on VOCs changes in TSB cultures (Filipiak et al. 2022). However, these studies employed gas chromatography–mass spectrometry (GC–MS), which has multiple pre-processing steps and long MS analysis time, making it unsuitable for widespread adoption. This calls for the development of more streamlined and rapid detection methods to facilitate early identification of CRKP.

Gas chromatography–ion mobility spectrometry (GC–IMS)—also referred to as gas electrophoresis (GEP) or plasma chromatography (PEC)—is a gas phase technique often used for the identification and characterization of VOCs. This technique leverages the differences in gas-phase ion mobility under a weak electric field, seamlessly merging the exceptional separation power of gas chromatography (GC) with the unmatched resolution, sensitivity, and accuracy of ion mobility spectrometry (IMS) (Drees et al. 2019). It has been widely investigated with regarding its ability to identify bacterial infections (Drees et al. 2019; Lacey et al. 2020; Lu et al. 2022).

In this study, we aimed to investigate differential VOCs between CSKP and CRKP using the GC–IMS method. VOCs were analyzed in simulated blood cultures (BCs) of both standards, under in vitro conditions, to identify metabolites associated with carbapenem resistance. The effects of imipenem (IPM) and CBPM inhibitors on VOCs were also examined.

## Materials and methods

### Bacteria strains and CBPM detection

The bacterial strains of *K. pneumoniae* ATCC BAA-1706 (CBPM-negative), ATCC BAA-1705 (*bla Klebsiella pneumoniae* carbapenemase (KPC)-positive), ATCC BAA-2146 (*bla* New Delhi metallo-β-lactamase (NDM)-positive), and ATCC BAA-2524 (*bla* oxacillinase-48 (OXA-48)-positive) were purchased from the American Type Culture Collection (ATCC) (Manassas, VA). Experiments involving these standard strains were performed in sextuplicate (one technical replicate was used in all samples).

Meanwhile, 69 *K. pneumoniae* isolates collected at the Second Affiliated Hospital of Nanchang University from January 1, 2016 to December 31, 2022 were analyzed. The isolates consisted of 25 CSKP isolates and 44 CRKP isolates (20 KPC-positive strains, 15 NDM-positive strains, 4 IPM-positive strains, and 5 CBPM-negative strains). The experiments involving these clinical strains were performed in triplicate for each strain.

All strains (standard and clinical strains) were kept in glycerol broth (15% glycerol) (Solarbio, China) in a − 80 °C freezer for further testing.

The identification of *K. pneumoniae* was conducted using the VITEK® 2 system (Bio-Merieux, Inc., France) or matrix-assisted laser desorption/ionization time-of-flight mass spectrometry (MALDI-TOF/MS) system (bioMérieux). Antibiotic susceptibility testing of *K. pneumoniae* was performed using a Kirby–Bauer (KB) test and the VITEK® 2 compact system, and the minimum inhibitory concentrations of ertapenem (ERT) and IPM were determined following the criteria set by the Clinical Laboratory Standards Institute (CLSI 2022). The assessment of CBPM production was conducted using two methodologies: the modified carbapenem inactivation method (mCIM) and the ethylenediaminetetraacetic acid (EDTA)-modified carbapenem inactivation method (eCIM). Moreover, the carbapenem resistance gene was characterized by polymerase chain reaction (PCR) amplification and sequencing.

### Schematic diagram of GC–IMS

VOCs in each sample group were detected using the commercial GC–IMS equipment (GC–IMS; FlavourSpec®; G.A.S., Dortmund, Germany). The integration of GC and IMS in GC–IMS enables the simultaneous achievement of high-resolution pre-separation (GC) and high-sensitivity detection (IMS). As described in Additional file 3: Fig. S1, initially, molecules from the sample are separated by the GC component based on their interactions with the stationary phase coating on the chromatographic column wall. Subsequent to the separation of molecules by GC, ionization typically occurs through a tritium source. The ionized molecules are then propelled along the drift tube under the influence of an electric field. Highly pure nitrogen gas (N_2_) introduced into the drift tube from the opposite direction causes molecules with varying mass and charge to exhibit different travel times to reach the Faraday plate. Finally, the combination of the retention time in the GC portion and the time of drift in the IMS section aids in the identification of compounds (Drees et al. 2019; Lu et al. 2022).

### Culture conditions and sample preparation

The culture solutions utilized for experimentation were acquired from BacT/ALERT® SA (Ref. 259789; Biomérieux, Nürtingen, Germany) BC bottles. The medium consisted of pancreatic digest of casein (1.7% w/v), papain digest of legume-based food (0.3% w/v), sodium polyanethole sulfonate (0.035% w/v), pyridoxine hydrochloride (0.001% w/v), and a combination of various amino acids, as well as hydrocarbon digests in purified water.

**Fig. 1:**
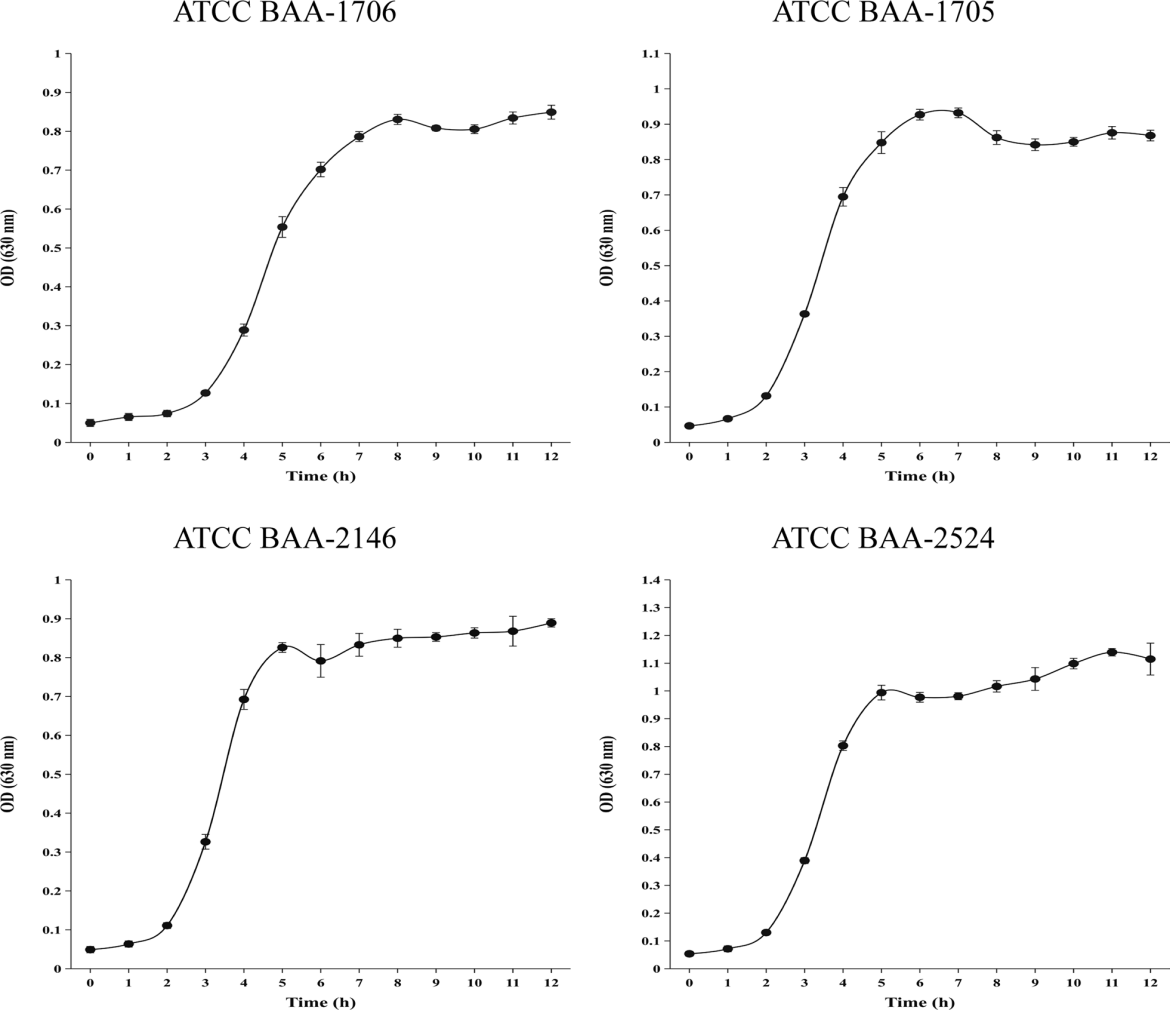
Growth curves of *K. pneumoniae* (standard strains)

As shown in Fig. 1, our preliminary findings demonstrated that the growth rate of *K. pneumoniae* exhibited its highest velocity at approximately 3 h after being subjected to the specified culture conditions (total volume: 6 mL; microbial concentration: 10^7^ colony forming units (CFU)/mL; culture medium: BacT/ALERT® SA; temperature: 37 °C; agitation: 200 rpm), subsequently transitioning into the end exponential growth phase around the 5 h mark.

The sample preparation process is presented in Additional file 3: Fig. S2. The experimental strains were inoculated on Columbia blood agar plates and incubated overnight at 37 °C. Subsequently, the bacterial suspensions were transferred to test tubes containing the culture medium (BacT/ALERT® SA), wherein the bacterial concentration was 10^7^ CFU/mL, with a total volume of 6 mL, and incubation was continued at 37 °C and 200 rpm agitation. About 500 mL of bacterial culture fluid for GC–IMS analysis was taken after incubation for 3 (T0), 4 (T1), 5 (T2), 6 (T3), and 7 (T4) h. A blank sterile medium served as a control.

To investigate the effect of IPM on CSKP and CRKP, a solution of IPM (Solarbio, China) was added to the bacterial suspension at the T0 time point, with a final concentration of 0.25 mg/mL (Luo et al. 2023). The incubation process was continued and all other conditions were maintained (Additional file 3: Fig. S2). Furthermore, subsequent experiments were undertaken to examine the influence of CBPM inhibitors on *K. pneumoniae* strains that produce CBPM. Similarly, the bacterial suspensions were simultaneously mixed with IPM and CBPM inhibitors [avibactam sodium or pyridine-2,6-dicarboxylic acid (DPA)] at T0, with avibactam sodium (Solarbio, China) concentration set at 1 mg/L (Luo et al. 2023) and DPA (Solarbio, China) concentration at 100 mg/L (Chen et al. 2017a).

### GC–IMS measurements

GC–IMS equipped with an MXT-WAX column (a high-polar column, 15 m × 0.53 mm, 0.1 μm, RESTEK, Bellefonte, PA, America) was used. The experimental parameters utilized in the GC–IMS analysis are displayed in Additional file 1: Table S1 and the schematic representation of the experimental workflow is depicted in Additional file 3: Fig. S3. Briefly, a headspace vial comprising the test culture medium (500 µL) was positioned within an autosampler and agitated at 500 rpm for 3 min at 60 °C. Subsequently, 1 mL of headspace gas was extracted from the vial, and the specimen under examination was introduced into the GC–IMS detection apparatus for 10 min.

### Data analysis

GC–IMS data were analyzed using the VOCal Version 0.1.3 software (G.A.S. mbH, Dortmund, Germany), employing C4–C9 ketones (2-butanone, 2-pentanone, 2-hexanone, 2-heptanone, 2-octanone, 2-nonanone) as reference standards. After data calibration, the VOCs were identified based on the retention index (RI) and drift time (reactant ion peak (RIP) relative) found in the GC–IMS library (NIST and IMS libraries) (Euler et al. 2022). Subsequently, the relative peak volume values (integrating peak intensities within specific regions after comparing calibrations) of VOCs were extracted from the software for statistical analysis. Given the limited number of VOCs (n = 54), potential VOCs exhibiting fold change (FC) values > 1.20 and a *P*-value < 0.05 were categorized as increased, whereas those with FC values < 0.83 and a *P*-value < 0.05 were categorized as decreased, in the context of inter-group comparisons (Mann–Whitney U test). Data processing and statistical analysis were conducted using R (version 4.2.1), while online tools ChiPlot (https://www.chiplot.online/) and OmicStudio (https://www.omicstudio.cn/tool), as well as Microsoft Office PowerPoint 2021 (Microsoft, Seattle, WA), were utilized for data visualization.

## Results

### Analysis of *K. pneumoniae* (standard strains) metabolites in the matrix components of BC bottles

A total of 54 VOCs (6 VOCs occurring as both monomers and dimers), including 4 organic acids, 3 alcohols, 3 esters, 4 ketones, 2 pyrazines, 2 benzene derivatives, and 30 unidentified compounds, were detected by GC–IMS (Table 1). Changes in the 54 VOCs throughout the time (T0–T4) were significant (Additional file 2: Table S2). *K. pneumoniae* reached the termination of its exponential growth phase at approximately 5 h (T2), and correspondingly, alterations in VOCs were no longer evident beyond T2 (Fig. 1). Hence, T2 was used for subsequent analysis.

**Table 1:** Information of the specific VOCs detected by GC–IMS

Chemical class	VOCs	CAS#	Formula	MW	RI	Rt [s]	Dt [a.u.]
Acids	3-Methylbutanoic acid^a^	C503742	C_5_H_10_O_2_	102.1	1750	586.33	1.22552
					1751	587.626	1.49209
	2-Methylpropanoic acid^a^	C79312	C_4_H_8_O_2_	88.1	1618.8	443.216	1.15693
					1617	441.548	1.37542
	Propionic acid^a^	C79094	C_3_H_6_O_2_	74.1	1587.2	414.364	1.11757
					1585.5	412.875	1.27296
	Acetic acid	C64197	C_2_H_4_O_2_	60.1	1484.4	332.819	1.16115
Alcohols	Butan-1-ol	C71363	C_4_H_10_O	74.1	1150.6	162.053	1.38774
	1,2-Ethanediol	C107211	C_2_H_6_O_2_	62.1	1699.3	526.24	1.17742
	3-Methyl-1-butanol	C123513	C_5_H_12_O	88.1	1212.2	186.46	1.49955
Esters	3-Methylbutyl propanoate	C105680	C_8_H_16_O_2_	144.2	1194.7	179.681	1.83371
	Ethyl acrylate	C140885	C_5_H_8_O_2_	100.1	1017.7	123.862	1.43157
	Acetic acid, ethyl ester	C141786	C_4_H_8_O_2_	88.1	851.1	97.222	1.10488
Ketones	Butan-2-one	C78933	C_4_H_8_O	72.1	921.8	107.351	1.25513
	Butane-2,3-dione (diacetyl)	C431038	C_4_H_6_O_2_	86.1	953.9	112.29	1.16815
	Isovalerone	C108838	C_9_H_18_O	142.2	1212	186.39	1.79551
	3-Hydroxy-2-butanone (acetoin)	C513860	C_4_H_8_O_2_	88.1	1304.2	226.606	1.33401
Pyrazines	2,5-Dimethylpyrazine^a^	C123320	C_6_H_8_N_2_	108.1	1339.9	244.561	1.11493
					1331.5	240.202	1.49283
	2-Methylpyrazine^a^	C109080	C_5_H_6_N_2_	94.1	1284.5	217.349	1.08982
					1282.2	216.283	1.38348
Benzene derivatives	Benzaldehyde^a^	C100527	C_7_H_6_O	106.1	1529.9	366.733	1.15758
					1531.1	367.685	1.47059
	Toluene	C108883	C_7_H_8_	92.1	1083.3	139.343	1.01594
Unidentified compounds	Unidentified-1	Unidentified	*	0	1666.5	490.716	1.10197
	Unidentified-2	Unidentified	*	0	1585.9	413.248	1.20044
	Unidentified-3	Unidentified	*	0	1328.8	238.801	1.39882
	Unidentified-4	Unidentified	*	0	1510.3	351.723	1.0557
	Unidentified-5	Unidentified	*	0	1233.6	195.132	1.05326
	Unidentified-6	Unidentified	*	0	1303.7	226.364	1.21631
	Unidentified-7	Unidentified	*	0	1306.5	227.705	1.37711
	Unidentified-8	Unidentified	*	0	1262.8	207.565	1.1872
	Unidentified-9	Unidentified	*	0	1224.5	191.396	1.37271
	Unidentified-10	Unidentified	*	0	1253.9	203.71	1.45577
	Unidentified-11	Unidentified	*	0	1320.8	234.806	1.03983
	Unidentified-12	Unidentified	*	0	1312.4	230.621	1.06936
	Unidentified-13	Unidentified	*	0	1289	219.402	1.0416
	Unidentified-14	Unidentified	*	0	1270	210.739	1.0412
	Unidentified-15	Unidentified	*	0	1229.9	193.581	1.27171
	Unidentified-16	Unidentified	*	0	1193.3	179.16	1.66092
	Unidentified-17	Unidentified	*	0	1180.1	173.703	1.48297
	Unidentified-18	Unidentified	*	0	1161.7	166.343	1.04652
	Unidentified-19	Unidentified	*	0	1159.4	165.457	1.18283
	Unidentified-20	Unidentified	*	0	1166.8	168.346	1.2179
	Unidentified-21	Unidentified	*	0	1146.9	160.645	1.44235
	Unidentified-22	Unidentified	*	0	1135.3	156.33	1.50645
	Unidentified-23	Unidentified	*	0	1109.1	146.938	1.46227
	Unidentified-24	Unidentified	*	0	1110.8	147.531	1.52464
	Unidentified-25	Unidentified	*	0	1125.8	152.855	1.24045
	Unidentified-26	Unidentified	*	0	1110	147.258	1.18923
	Unidentified-27	Unidentified	*	0	1042.1	129.396	1.19766
	Unidentified-28	Unidentified	*	0	885.9	102.081	1.19145
	Unidentified-29	Unidentified	*	0	833.1	94.795	1.46465
	Unidentified-30	Unidentified	*	0	793.9	89.728	1.1149

*GC–IMS* gas chromatography–ion mobility spectrometry, *VOCs* volatile organic compounds, *CAS#* Chemical Abstract Service Registry Number, *MW* molecular weight, *RI* retention index, *Rt* retention time, *Dt* drift time

^a^Represents the substance has two distinct peak positions in the GC–IMS system, with a shorter drift time corresponding to the monomer and a longer drift time corresponding to the dimer. “*” Represents the formula of VOC is unknown

**Fig. 2:**
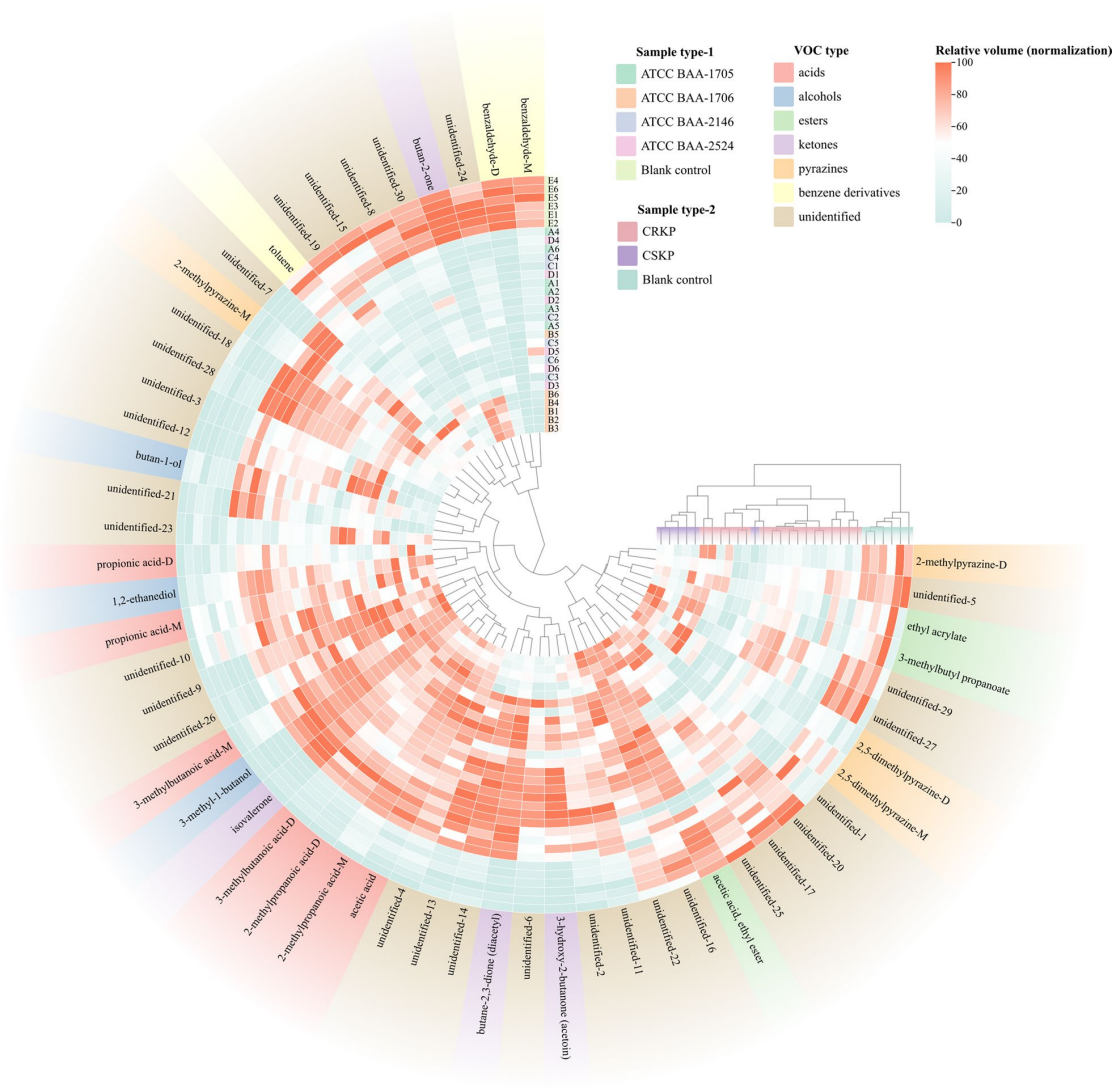
The heatmap of VOCs expression profile of each sample (after min–max normalization)

**Fig. 3:**
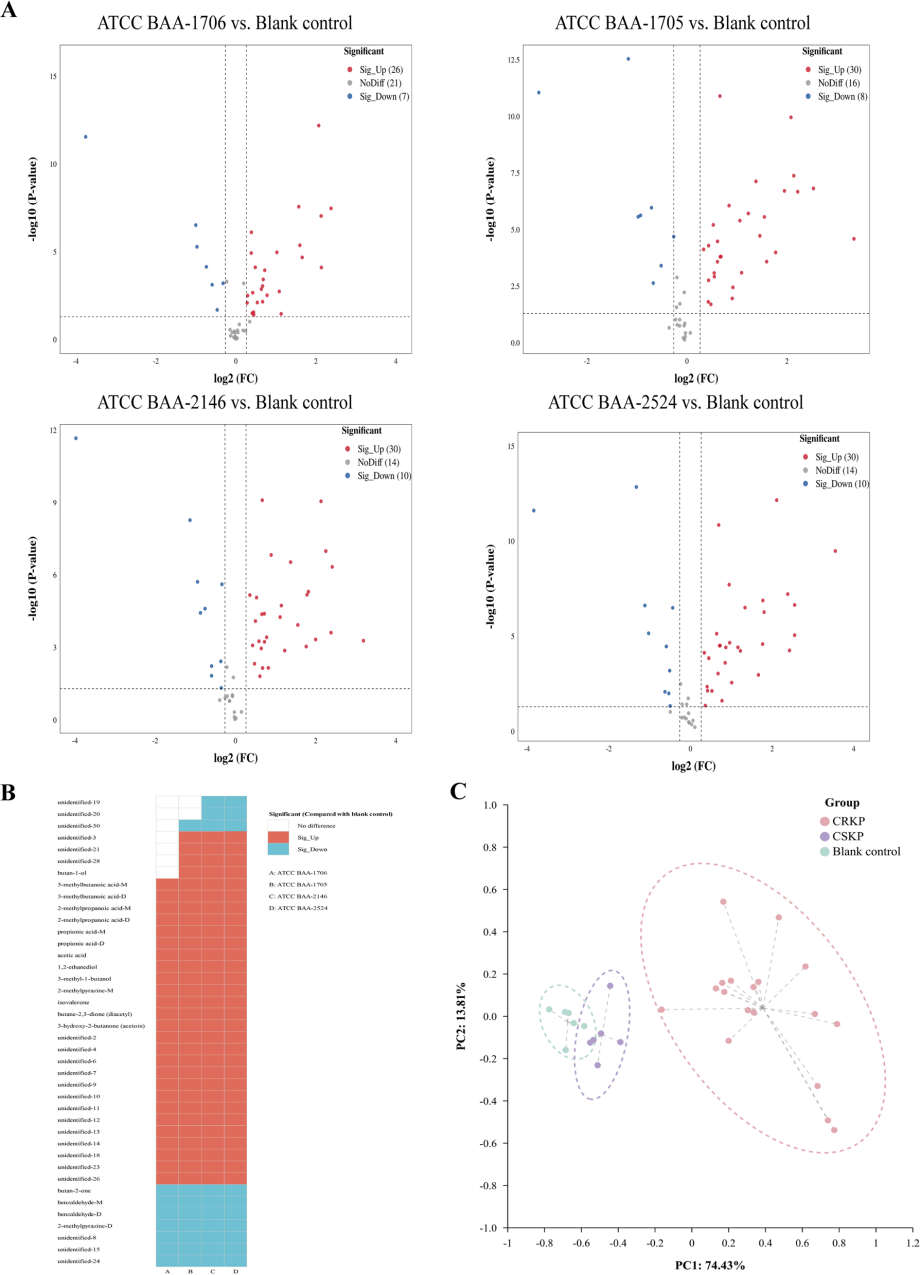
The VOCs emitted by *K. pneumoniae* (standard strains, without IPM added). **A** The volcano plots of differential VOCs between the blank control group and the experimental groups. **B** The heatmap of the differentially expressed VOCs determined using the volcano plots (compared with the blank control group, after binarization). **C** The PCA of the sample groups (without IPM added)

Changes in VOCs for each of the standard strains (ATCC BAA-1706, ATCC BAA-1705, ATCC BAA-2146, and ATCC BAA-2524) compared with the blank control group at T2 are shown in Fig. 2 and Additional file 2: Table S3. Compared with the blank control group, 26 VOCs were increased whereas 7 were decreased in the ATCC BAA-1706. For *K. pneumoniae* ATCC BAA-1705, 30 VOCs were increased whereas 8 were decreased. For *K. pneumoniae* ATCC BAA-2146, 30 VOCs were increased and 10 were decreased, and 30 VOCs were increased and 10 were decreased in the *K. pneumoniae* ATCC BAA-2524 (Fig. 3A).

Further analysis demonstrate that the principal VOCs released by *K. pneumoniae* (all standard strains) metabolites in the matrix components of BC bottles encompassed organic acids (3-methylbutanoic acid (monomer and dimer), 2-methylpropanoic acid (monomer and dimer), propionic acid (monomer and dimer), and acetic acid), alcohols (1,2-ethanediol and 3-methyl-1-butanol), ketones [isovalerone, butane-2,3-dione (diacetyl), and 3-hydroxy-2-butanone (acetoin)], and 2-methylpyrazine-M (monomer), compared with the blank control group. Meanwhile, benzaldehyde (monomer and dimer), butan-2-one, and 2-methylpyrazine-D (dimer) in *K. pneumoniae* standard strains were significantly reduced compared with those in the blank control group (Fig. 3B and Table 2). However, 13 VOCs exhibiting an upward trend and 3 VOCs displaying a downward trend were not accurately identified (Fig. 3B and Table 2).

**Table 2:** Volatile metabolic profiles of *Klebsiella pneumoniae* (standard strains) in the matrix components of BC bottles (at T2 time point)

Class	VOCs	CAS#	Blank control (n = 6)	ATCC BAA-1706 (n = 6)		ATCC BAA-1705 (n = 6)		ATCC BAA-2146 (n = 6)		ATCC BAA-2524 (n = 6)	
			Relative volume	Relative volume	Variation	Relative volume	Variation	Relative volume	Variation	Relative volume	Variation
Unidentified	Unidentified-19	*	259.36 ± 27.03	243.31 ± 34.41	−	221.85 ± 41.66	–	201.35 ± 26.31	Taken up	172.26 ± 13.53	Taken up
	Unidentified-20	*	80.46 ± 13.71	77.51 ± 14.68	–	70.26 ± 9.21	–	53.26 ± 17.94	Taken up	56.99 ± 21.07	Taken up
	Unidentified-30	*	3719 ± 125.19	3621.35 ± 198.77	–	3096.39 ± 159.97	Taken up	2944.65 ± 154.04	Taken up	2751.87 ± 155.1	Taken up
	Unidentified-3	*	24.58 ± 6.89	31.23 ± 5.5	–	95.15 ± 11.95	Released	98.25 ± 34.74	Released	132.08 ± 38.88	Released
	Unidentified-21	*	59.13 ± 9.87	60.45 ± 9.86	–	110.81 ± 39.38	Released	94.55 ± 23.69	Released	106.63 ± 18.48	Released
	Unidentified-28	*	1621.2 ± 80.95	1855.21 ± 84.58	–	2337.44 ± 187.02	Released	2180.71 ± 278.99	Released	2216.02 ± 230.88	Released
Alcohol	Butan-1-ol	C71363	73.24 ± 16.55	86.74 ± 27.29	–	138.6 ± 38.88	Released	111.8 ± 27.77	Released	133.57 ± 13.26	Released
Acids	3-Methylbutanoic acid-M	C503742	1098.73 ± 238.8	1810.91 ± 154.94	Released	1742.93 ± 124.07	Released	1814.19 ± 261.77	Released	1756.62 ± 249.92	Released
	3-Methylbutanoic acid-D	C503742	101.36 ± 29.41	307.2 ± 47.79	Released	296.94 ± 41.48	Released	349.19 ± 64.56	Released	353.99 ± 46.52	Released
	2-Methylpropanoic acid-M	C79312	568.33 ± 106.22	912.56 ± 120.34	Released	868 ± 80.78	Released	936.64 ± 75.1	Released	931.79 ± 65.31	Released
	2-Methylpropanoic acid-D	C79312	34.52 ± 9.81	108.93 ± 22.23	Released	94.98 ± 16.88	Released	121.88 ± 22.14	Released	118.1 ± 12.23	Released
	Propionic acid-M	C79094	323.07 ± 79.68	467.81 ± 70.81	Released	434.6 ± 49.44	Released	504.7 ± 57.62	Released	416.13 ± 57.95	Released
	Propionic acid-D	C79094	153.2 ± 62.49	335.66 ± 170.78	Released	327.15 ± 64.73	Released	358.99 ± 96.18	Released	308.82 ± 73.07	Released
	Acetic acid	C64197	1895.29 ± 460.5	3853.23 ± 372.54	Released	3951.13 ± 314.38	Released	4191.02 ± 578.87	Released	4788.16 ± 376.58	Released
Alcohols	1,2-Ethanediol	C107211	433.73 ± 80.85	673.84 ± 106.2	Released	633.41 ± 65.03	Released	651.28 ± 70.04	Released	579.91 ± 68.98	Released
	3-Methyl-1-butanol	C123513	365.1 ± 28.6	1535.16 ± 56.1	Released	1547.09 ± 103.04	Released	1602.3 ± 135.29	Released	1578.97 ± 59.39	Released
Pyrazine	2-Methylpyrazine-M	C123320	417.89 ± 46.44	512.32 ± 37.54	Released	564.31 ± 70.81	Released	590.59 ± 47.41	Released	554.8 ± 78.91	Released
Ketones	Isovalerone	C108838	35.98 ± 9.34	187.19 ± 22.71	Released	208.29 ± 31.43	Released	191.7 ± 32.02	Released	210.91 ± 33.45	Released
	Butane-2,3-dione (diacetyl)	C431038	2731.93 ± 61.59	3574.11 ± 180.22	Released	4318.89 ± 97.6	Released	4340.92 ± 166.94	Released	4416.61 ± 107.41	Released
	3-Hydroxy-2-butanone (acetoin)	C513860	117.27 ± 9.05	515.91 ± 152.06	Released	1190.89 ± 359.11	Released	1071 ± 465.59	Released	1373.15 ± 126.62	Released
Unidentified	Unidentified-2	*	361.62 ± 69.1	491.72 ± 102.36	Released	580.59 ± 59.46	Released	620.14 ± 99.15	Released	704.14 ± 88.99	Released
	Unidentified-4	*	3066.57 ± 358.67	4289.56 ± 297.7	Released	4142.61 ± 155.48	Released	4415.25 ± 173.28	Released	4787.3 ± 347.86	Released
	Unidentified-6	*	88.41 ± 14.03	140.42 ± 34.89	Released	267.38 ± 78.32	Released	259.91 ± 67.49	Released	301.01 ± 69.92	Released
	Unidentified-7	*	125.84 ± 13.48	374.06 ± 37.01	Released	431.21 ± 119.88	Released	427.64 ± 158.59	Released	398.99 ± 146.19	Released
	Unidentified-9	*	32.41 ± 4.15	141.95 ± 19.29	Released	142.74 ± 17.86	Released	154.49 ± 21.9	Released	169.15 ± 23.28	Released
	Unidentified-10	*	79.01 ± 17.28	104.81 ± 18.32	Released	110.23 ± 21.57	Released	109.93 ± 11.7	Released	113.83 ± 18.69	Released
	Unidentified-11	*	100.71 ± 16.36	134.81 ± 12.06	Released	154.04 ± 8.43	Released	159 ± 12.68	Released	166.61 ± 15.56	Released
	Unidentified-12	*	21.19 ± 5.45	44.93 ± 12.68	Released	98.59 ± 14.26	Released	110.49 ± 39.04	Released	124.2 ± 30.01	Released
	Unidentified-13	*	44.24 ± 7.5	60.28 ± 14.7	Released	115.26 ± 10.05	Released	114.78 ± 12.28	Released	103.3 ± 20.5	Released
	Unidentified-14	*	166.02 ± 19.43	201.93 ± 18.3	Released	298.01 ± 23.2	Released	307.66 ± 18.58	Released	320.56 ± 13.62	Released
	Unidentified-18	*	48.85 ± 9.46	77.54 ± 11.72	Released	114.47 ± 13.42	Released	105.71 ± 18.6	Released	109.41 ± 19.06	Released
	Unidentified-23	*	77.24 ± 16.5	132.65 ± 30.64	Released	112.99 ± 10.54	Released	136.41 ± 39.4	Released	131.93 ± 47.65	Released
	Unidentified-26	*	892.84 ± 24.01	1164.59 ± 79.53	Released	1126.08 ± 85.41	Released	1145.93 ± 68.69	Released	1129.47 ± 86.7	Released
Ketone	Butan-2-one	C78933	3747.72 ± 80.94	3000.6 ± 363.77	Taken up	1662.6 ± 64.71	Taken up	1708.1 ± 261.89	Taken up	1497.46 ± 65.5	Taken up
Benzene derivatives	Benzaldehyde-M	C100527	2056.24 ± 157.16	1025.85 ± 140.35	Taken up	1257.39 ± 102.26	Taken up	1216.34 ± 231.92	Taken up	1445.36 ± 262.24	Taken up
	Benzaldehyde-D	C100527	3621.7 ± 205.4	268.03 ± 40.72	Taken up	463.75 ± 82.89	Taken up	230.02 ± 18.91	Taken up	253.73 ± 29.47	Taken up
Pyrazine	2-Methylpyrazine-D	C123320	165.83 ± 20.54	109.9 ± 19.98	Taken up	115.93 ± 11.25	Taken up	129.81 ± 32.98	Taken up	114.86 ± 33.52	Taken up
Unidentified	Unidentified-8	*	339.64 ± 34.05	203.53 ± 38.65	Taken up	178.48 ± 23.2	Taken up	176.38 ± 22.47	Taken up	156.91 ± 13.24	Taken up
	Unidentified-15	*	68.64 ± 13.42	49.61 ± 10.27	Taken up	43.01 ± 7.7	Taken up	45.47 ± 9.13	Taken up	44.52 ± 11.9	Taken up
	Unidentified-24	*	468.99 ± 48.44	238.66 ± 42.14	Taken up	238.43 ± 35.16	Taken up	256.3 ± 56.44	Taken up	230.74 ± 48.84	Taken up

“–” Represents no statistically significant variance when compared to the blank control group in terms of absorption or release by *Klebsiella pneumoniae* based on our established criteria. “*” Represents the CAS# of VOC is unknown

*VOCs* volatile organic compounds, *CAS#* Chemical Abstract Service Registry Number, *M* monomer, *D* dimer

### The utility of VOCs in identifying CRKP based on standard strains

Next, *K. pneumoniae* ATCC BAA-1706 (CBPM-negative) was used as a negative control (CSKP), and *K. pneumoniae* ATCC BAA-1705 (*bla* KPC-positive), *K. pneumoniae* ATCC BAA-2146 (*bla* NDM-positive), *K. pneumoniae* ATCC BAA-2524 (*bla* OXA-48-positive) were used as a positive control (CRKP).

To investigate the potential of VOCs in distinguishing CRKP, a comprehensive analysis was conducted to examine the disparities in VOCs between CSKP and CRKP. Remarkably, 5 VOCs (unidentified-3, unidentified-21, unidentified-28, and butan-1-ol were increased, whereas unidentified-30 was decreased) differed significantly in the CRKP group compared with the CSKP group (Fig. 3B and Additional file 3: Fig. S4A). Subsequently, principal component analysis (PCA) showed that the 5 VOCs were effective in distinguishing between CSKP and CRKP in the T2 time point (Fig. 3C). Intriguingly, these differences disappeared with time (Additional file 3: Fig. S4B).

### Differential VOCs between CSKP and CRKP (standard strains) after the addition of IPM

**Fig. 4:**
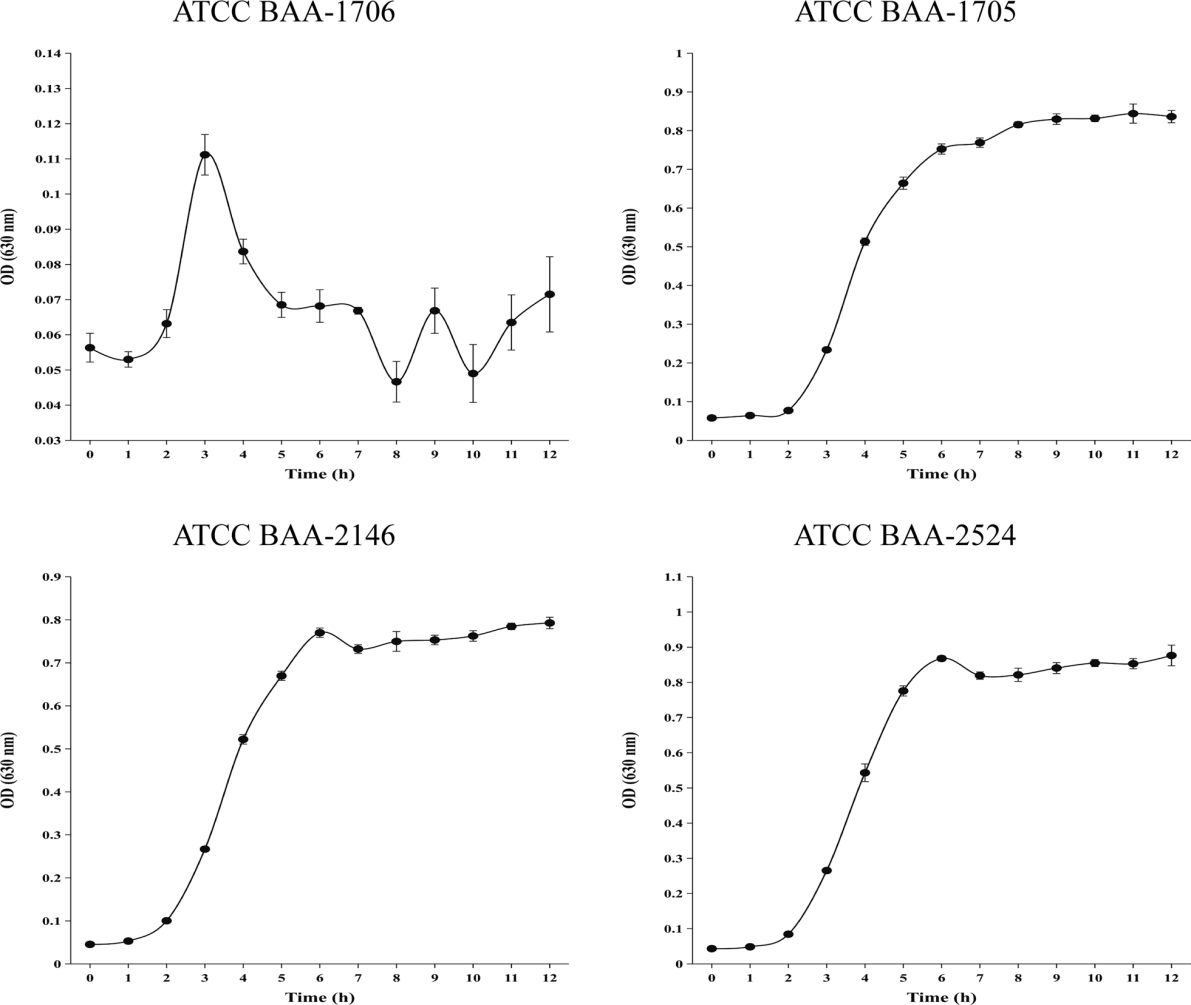
The growth curves of *K. pneumoniae* standard strains after the addition of imipenem at time T0 (3 h of bacteria growth)

To assess the impact of IPM on VOC emission from *K. pneumoniae*, IPM was added to the test tubes at T0 to a concentration of 0.25 mg/mL (Additional file 3: Fig. S2). Subsequent growth curve analyses demonstrated that the introduction of IPM after a 3 h incubation period exclusively affects the growth of CBMP-negative strain (the bacteria were killed completely), while CBMP-positive strains exhibited a consistent growth pattern comparable to that observed in the absence of IPM supplementation (Figs. 1 and 4).

It was found that the introduction of IPM did not yield any new VOCs. However, notable alterations were observed in the content of certain VOCs of *K. pneumoniae* ATCC BAA-1706 in the T2 time point (after 2 h of IPM addition), which was maintained until the end of the study (Additional file 2: Table S4). Concurrently, VOCs of *K. pneumoniae* ATCC BAA-1705, ATCC BAA-2146, and ATCC BAA-2524 showed similar temporal change trends to those without IPM addition (Additional file 2: Table S4).

**Fig. 5:**
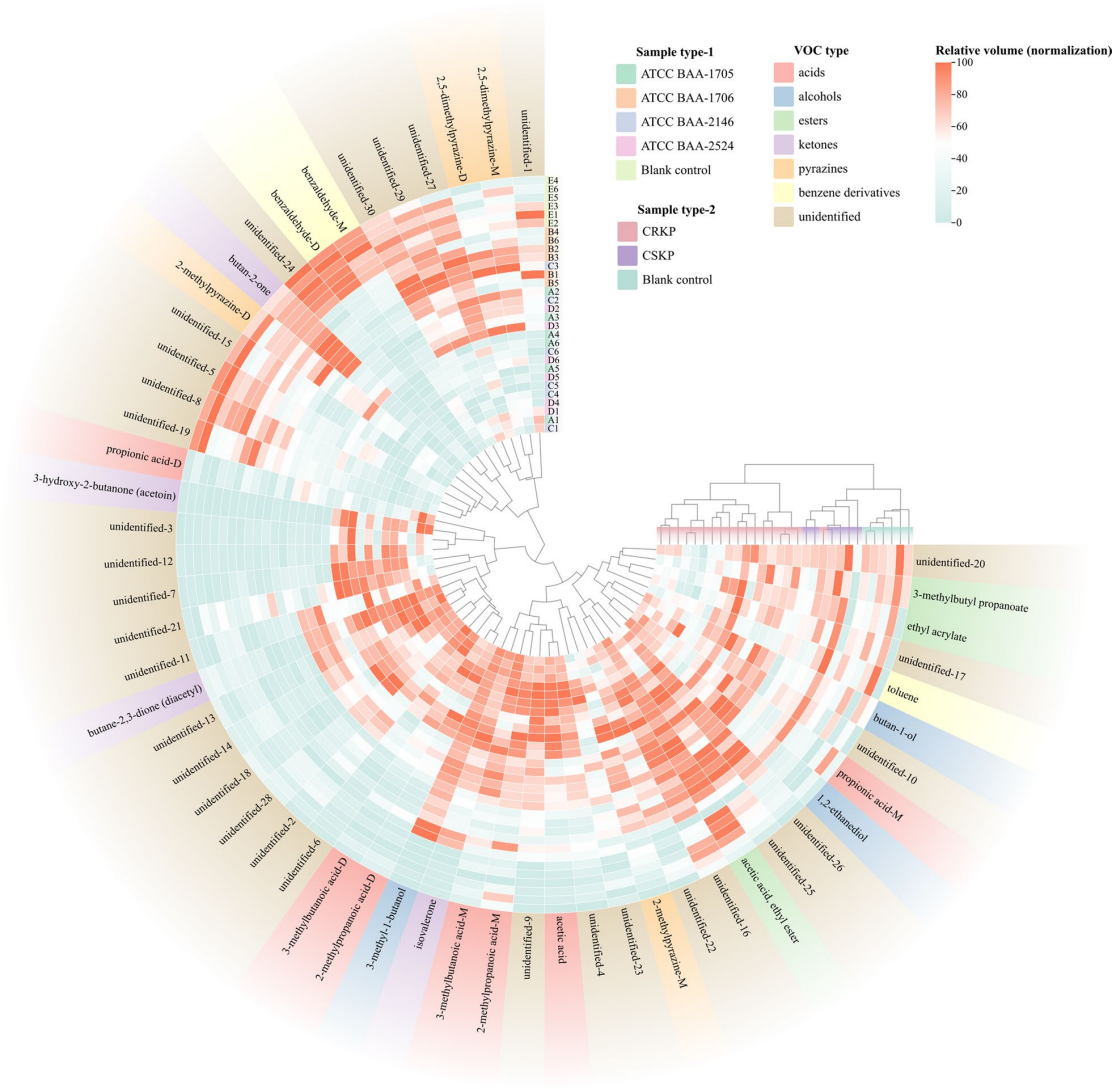
The heatmap of the VOCs expression in all samples (treated with IPM, after min–max normalization)

**Fig. 6:**
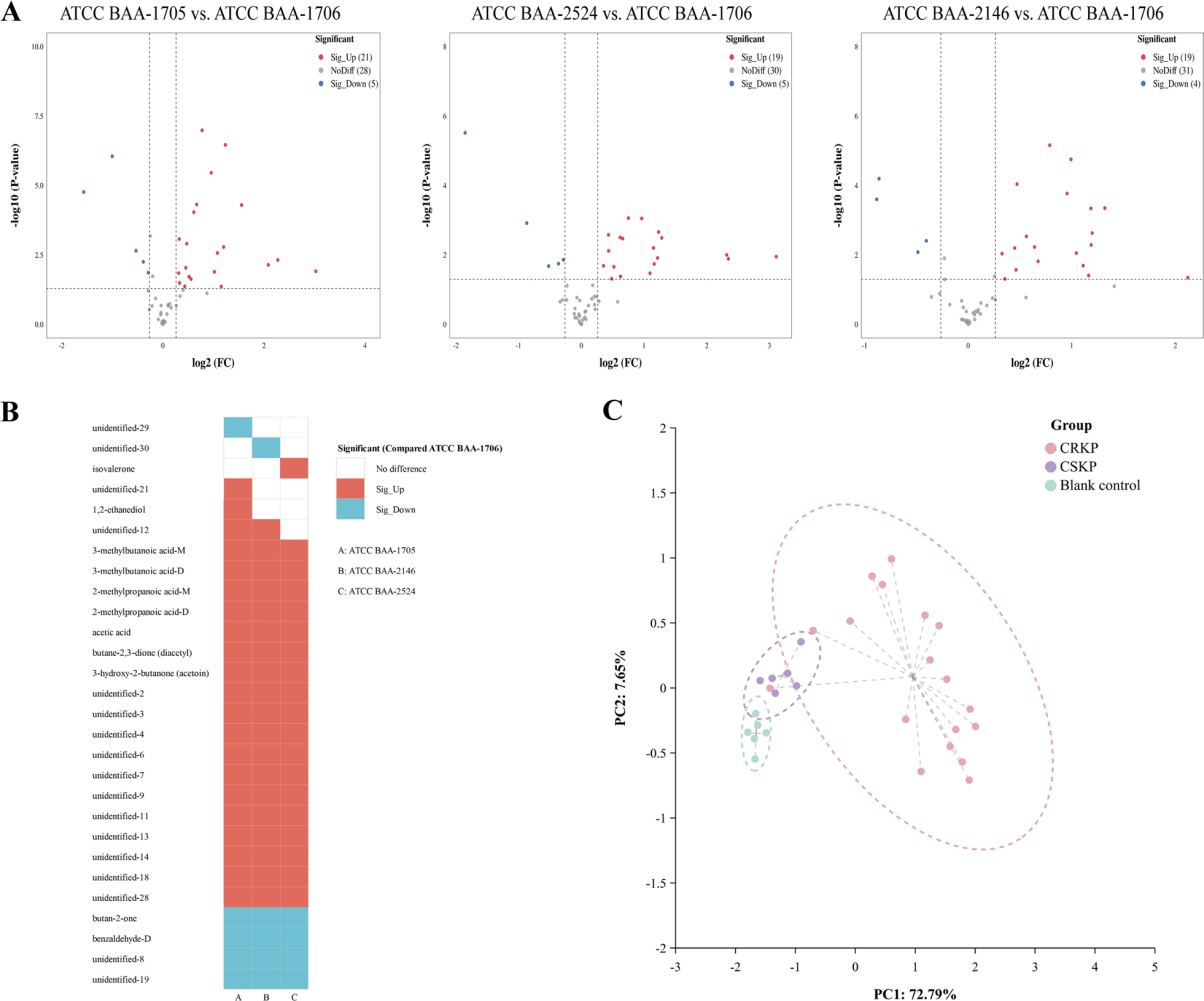
The VOCs emitted by *K. pneumoniae* (standard strains, with IPM added). **A** Volcano plots of differential VOCs between the CSKP group and CRKP strains. **B** The heatmap of the differentially expressed VOCs generated using volcano plots (compared with the CSKP group, after binarization). **C** The PCA of various sample groups (treated with IPM)

Further analysis revealed that compared with *K. pneumoniae* ATCC BAA-1706, *K. pneumoniae* ATCC BAA-1705 displayed a significant divergence of 26 VOCs (21 VOCs were increased and 5 were decreased), *K. pneumoniae* ATCC BAA-2146 showed a substantial variation of 24 VOCs (19 VOCs were increased and 5 were decreased), and *K. pneumoniae* ATCC BAA-2524 exhibited a marked distinction of 23 VOCs (19 VOCs were increased and 4 were decreased) at the T2 time point (Figs. 5 and 6A and Additional file 2: Table S5).

After a comprehensive analysis of these VOCs, the contents of 3-methylbutanoic acid (monomer and dimer), 2-methylpropanoic acid (monomer and dimer), acetic acid, butane-2,3-dione (diacetyl), and 3-hydroxy-2-butanone (acetoin) were significantly higher in the CRKP group than in the CSKP group, while the contents of benzaldehyde-D and butan-2-one were significantly lower. Meanwhile, 11 VOCs exhibiting an upward trend and 2 VOCs displaying a downward trend were not accurately identified (Fig. 6B). As shown in Additional file 3: Fig. S5, significant correlations were observed among the 22 increased VOCs and 6 decreased VOCs in the CRKP group. PCA further revealed that these differential VOCs were effective in discriminating between CRKP and CSKP (Fig. 6C). Interestingly, these differences persisted from T2 to T4 (Additional file 2: Table S4 and Additional file 3: Fig. S6).

### Potential utility of VOCs in identifying CBPM-producing *K. pneumoniae* based on standard strains

To more comprehensively evaluate the significance of VOCs in the identification of CBPM-producing *K. pneumoniae* and determine its phenotypes, both IPM (final concentration, 0.25 mg/mL) and CBPM inhibitors (final concentration: avibactam sodium, 1 mg/L; or DPA, 100 mg/L) were added into the test tubes after a 3-h incubation period (T0). Moreover, changes in VOCs were assessed at the T2 time point before and after the addition of enzyme inhibitors.

**Fig. 7:**
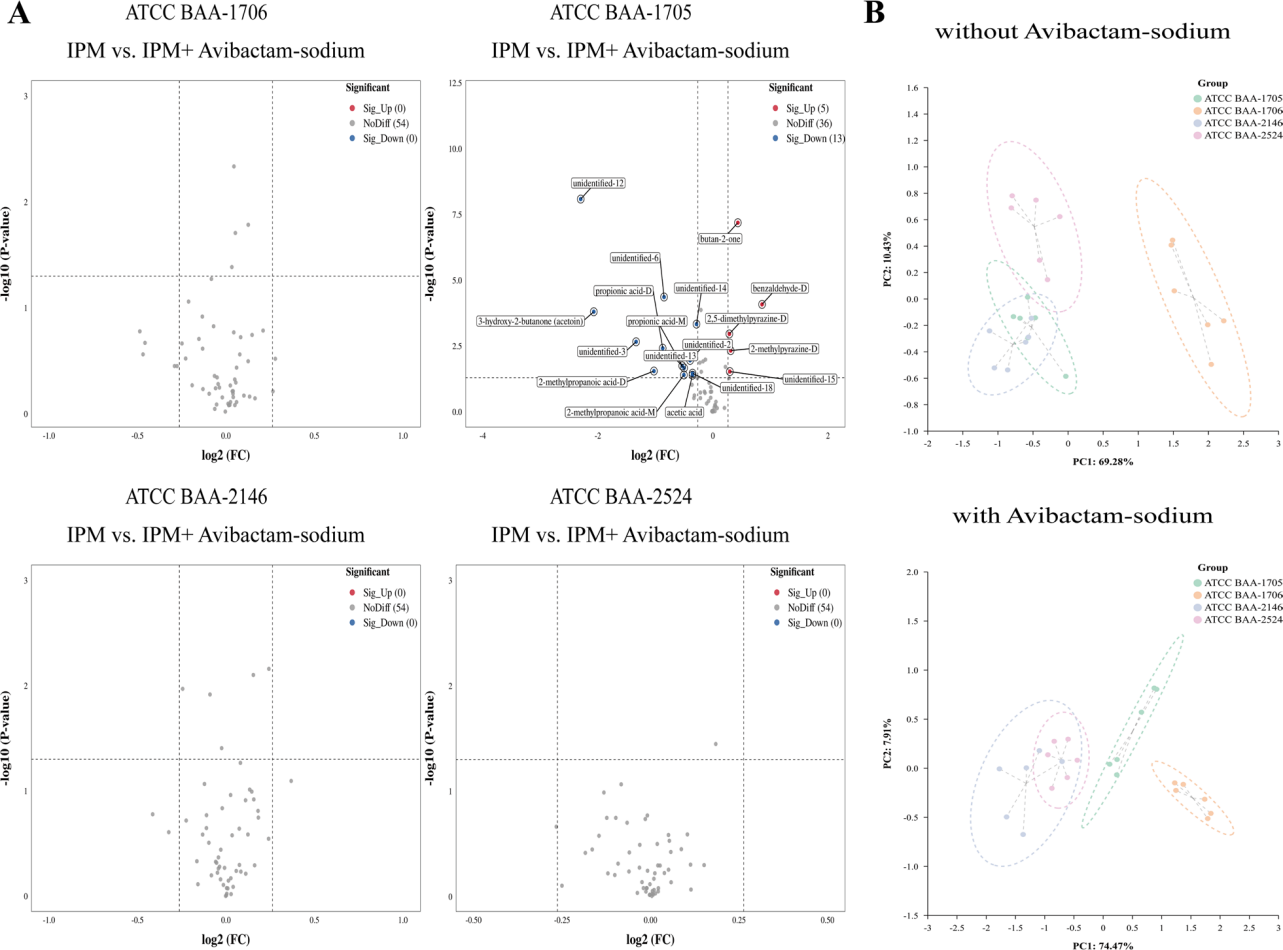
The VOCs emitted by *K. pneumoniae* after addition of avibactam sodium (standard strains, with IPM added). **A** The volcano plots displaying the differential VOCs between the pre- and post-inhibitor (avibactam sodium) addition stages for each strain. **B** The PCA of various sample groups (avibactam sodium unspiked and avibactam sodium spiked)

**Fig. 8:**
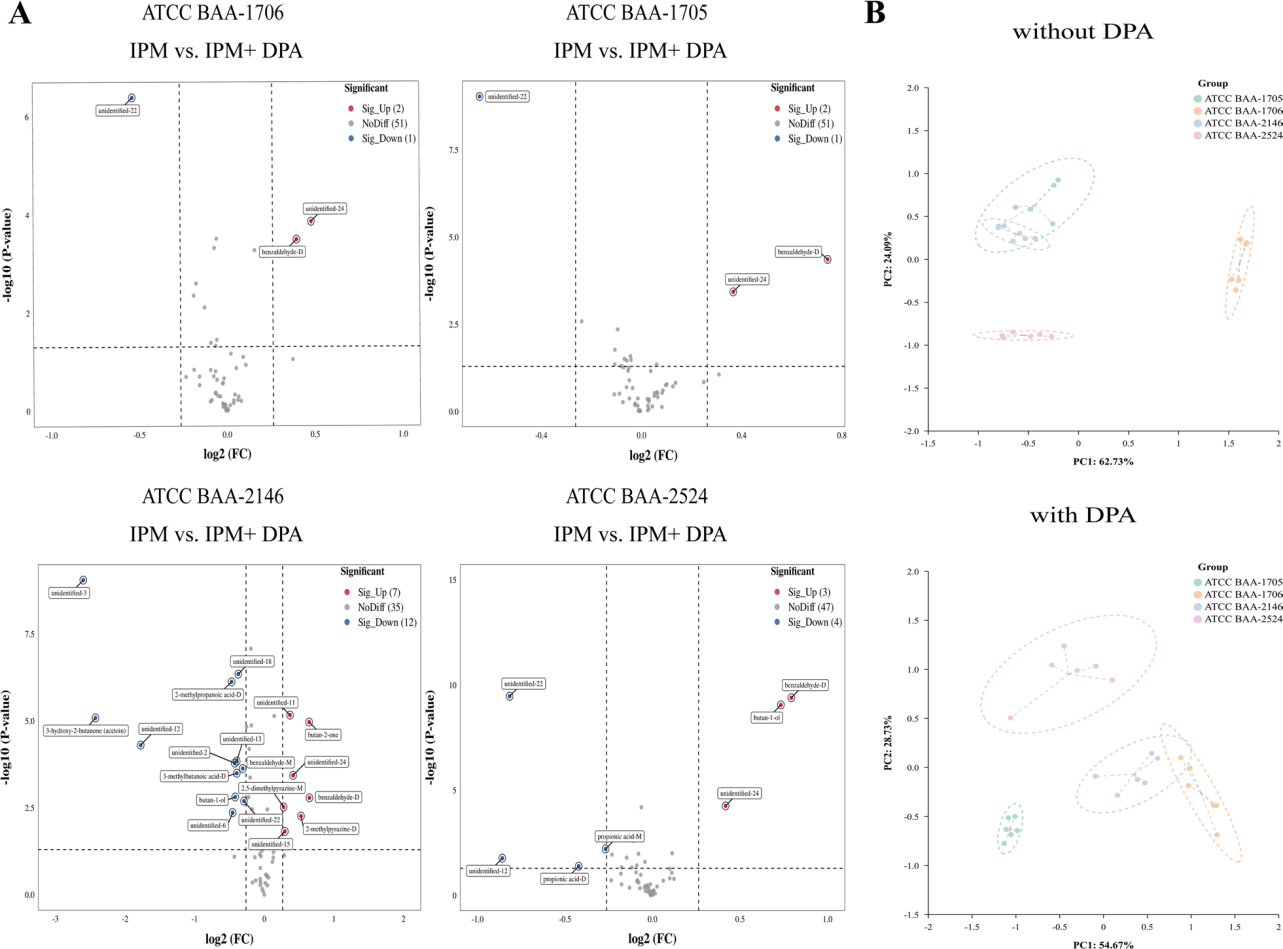
The VOCs emitted by *K. pneumoniae* after addition of DPA (standard strains, with IPM added). **A** Volcano plots illustrating the differential VOCs between the pre- and post-inhibitor (DPA) addition stages for each strain. **B** The PCA of the sample groups (DPA unspiked and DPA spiked)

Changes in the VOCs of the standard strains after the addition of CBPM inhibitors compared with those without CBPM inhibitors are presented in Additional file 2: Table S6. Results showed that the addition of avibactam sodium resulted in changes in the content of certain VOCs exclusively in *K. pneumoniae* ATCC BAA-1705, including 5 increased VOCs (benzaldehyde-D, 2,5-dimethylpyrazine-D, 2-methylpyrazine-D, butan-2-one, and unidentified-15) and 13 decreased VOCs (2-methylpropanoic acid (monomer and dimer), propionic acid (monomer and dimer), acetic acid, 3-hydroxy-2-butanone (acetoin), and 7 unidentified VOCs) (Fig. 7A). PCA results revealed that the addition of avibactam sodium lead to the identification of class A CBPM-producing *K. pneumoniae* (*K. pneumoniae* ATCC BAA-1705) via changes in VOCs (Fig. 7B). Similarly, the addition of DPA exerted the most significant influence on the contents of some VOCs in *K. pneumoniae* ATCC BAA-2146, including 5 increased VOCs (2,5-dimethylpyrazine-M, 2-methylpy,razine-D, butan-2-one and 2 unidentified VOCs) and 10 decreased VOCs (3-methylbutanoic acid-D, 2-methylpropanoic acid-D, benzaldehyde-M, 3-hydroxy-2-butanone (acetoin), butan-1-ol and 5 unidentified VOCs) (Fig. 8A). PCA further showed that the 15 differential VOCs after the addition of DPA were effective in identifying class B CBPM-producing *K. pneumoniae* (*K. pneumoniae* ATCC BAA-2146) (Fig. 8B). Additionally, Additional file 3: Fig. S7 confirmed that this experiment was repeated six times with high reproducibility.

### Potential application of VOCs in the identification of CRKP in clinical strains after the addition of IPM

The growth time curve of clinically isolated strains is shown in Additional file 3: Fig. S8, which was in line with the growth curves of standard strains. Using the T2 time point as the focal point, we further examined the disparity in VOCs between CSKP and CRKP.

The follow-up study encompassed 69 *K. pneumoniae* clinical isolates, comprising 25 CSKP strains and 44 CRKP strains. Experiments were performed in triplicates for each clinical isolate at the T2 time point utilizing GC–IMS. Moreover, the 44 CRKP strains included 20 KPC-positive strains, 15 NDM-positive strains, 4 IPM-positive strains, and 5 CBPM-negative strains. Given that CBPM-negative strains could not withstand a high concentration of IPM (0.25 mg/mL), the final concentration of IPM after a 3-h incubation period (T0) in the matrix components of BC bottles was adjusted to 16 µg/mL.

**Fig. 9:**
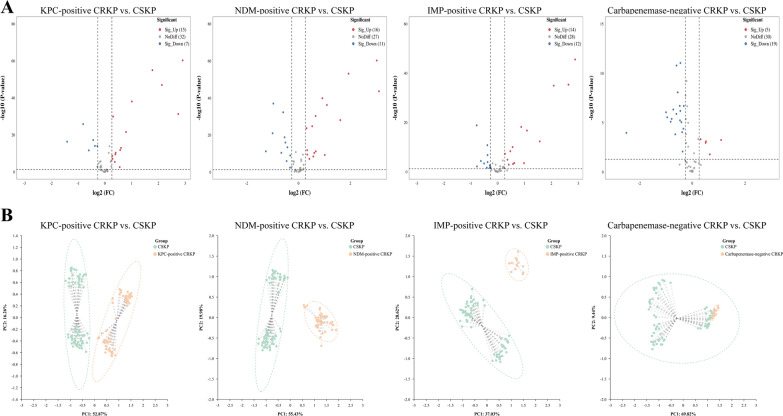
The VOCs produced by *K. pneumoniae* (clinical strains, with IPM added). **A** The volcano plots of differential VOCs between the CSKP group and CRKP strains. **B** The PCA of the sample groups (with IPM added)

Changes in VOCs emitted by CRKP and CSKP at T2 are shown in Additional file 2: Table S7. Compared with the CSKP group, KPC-positive strains exhibited a notable disparity of 22 VOCs (15 increased and 7 decreased VOCs), NDM-positive strains displayed a significant divergence of 27 VOCs (16 increased and 11 decreased VOCs), IPM-positive strains demonstrated a substantial variation of 26 VOCs (14 increased and 12 decreased VOCs), and CBPM-negative strains had a marked distinction of 24 VOCs (5 increased and 19 decreased VOCs) (Fig. 9A and Additional file 3: Fig. S9). Further analysis of PCA results indicated that the differential VOCs with the addition of IPM were effective in identifying CBPM*-*producing *Klebsiella pneumoniae* (KPC-positive, NDM-positive, and IMP-positive strains); however, differential VOCs could not effectively distinguish between CSKP and CBPM-negative strains (Fig. 9B).

### Potential utility of VOCs in identifying CBPM-producing *K. pneumoniae* based on clinical strains

Next, we investigated the importance of VOCs in identifying CBPM-producing *K*. *pneumoniae* and its phenotypes in clinical strains, following the same manipulation procedures as those applied to standard strains (CBPM-negative strains: IPM, final concentration, 16 µg/mL; avibactam sodium, 1 mg/L; DPA, 100 mg/L). Experiments were performed in triplicates for each clinical isolate at the T2 time point utilizing GC–IMS. Furthermore, considering that previous studies have shown that differential VOCs may differ between CSKP and CBPM-producing *K. pneumoniae*, changes in VOCs emitted by CSKP strains following the addition of CBPM inhibitors were not explored further.

**Fig. 10:**
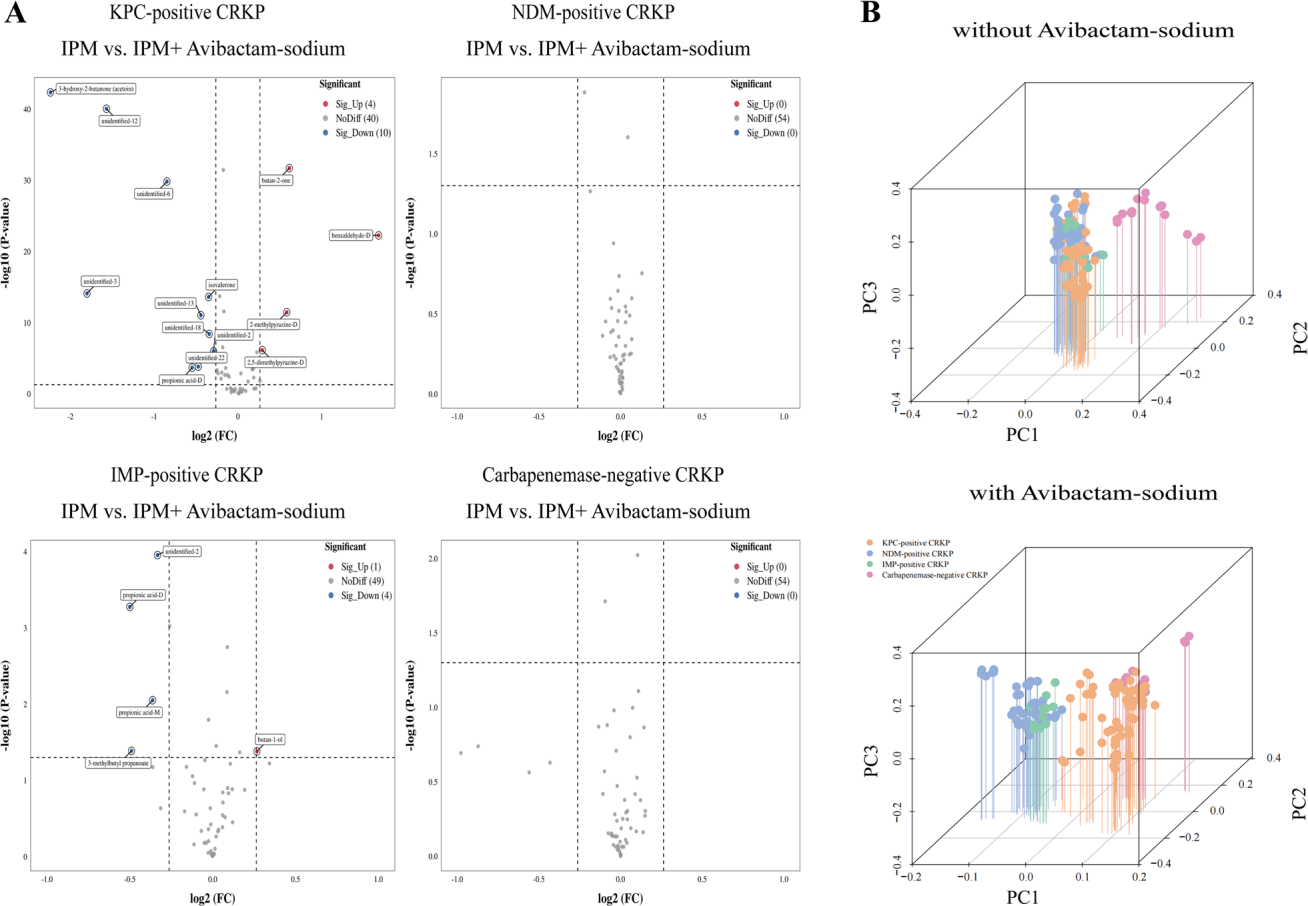
The VOCs produced by *K. pneumoniae* after addition of avibactam sodium (clinical strains, with IPM added). **A** Volcano plots illustrating the differential VOCs between the pre- and post-inhibitor (avibactam sodium) addition stages for each strain. **B** The PCA of the sample groups (avibactam sodium unspiked and avibactam sodium spiked)

**Fig. 11:**
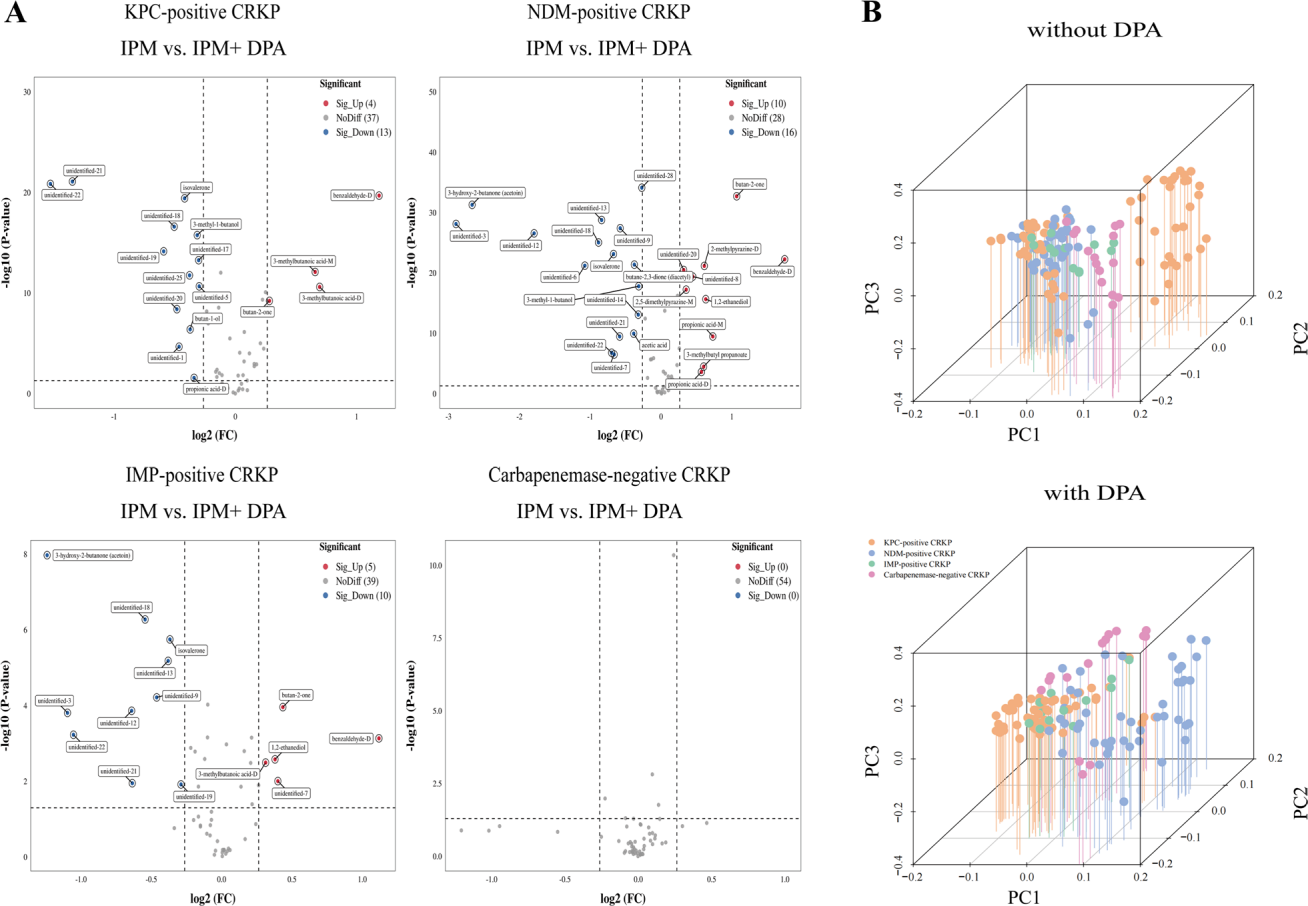
The VOCs produced by *K. pneumoniae* after the addition of DPA (clinical strains, with IPM added). **A** The volcano plots illustrating the differential VOCs between the pre- and post-inhibitor (DPA) addition stages for each strain. **B** The PCA of the sample groups (DPA unspiked and DPA spiked)

Changes in VOCs emitted by CRKP strains after addition of CBPM inhibitors are shown in Additional file 2: Table S8. After the addition of avibactam sodium, only KPC-positive strains showed changes in the content of some VOCs, including 4 increased VOCs (benzaldehyde-D, 2,5-dimethylpyrazine-D, 2-methylpyrazine-D, and butan-2-one) and 8 decreased VOCs (3-hydroxy-2-butanone (acetoin), isovalerone, and 6 unidentified VOCs) (Fig. 10A). Moreover, a separation trend was observed in a three-dimensional PCA score plot after including the 12 potential biomarker VOCs, which were effective in identifying class A CBPM-producing *K. pneumoniae* (KPC-positive strains) after the addition of avibactam sodium (Fig. 10B). Subsequently, after the addition of DPA, there were significant changes in some VOCs, including 8 increased VOCs and 11 decreased VOCs in the NDM-positive strains. However, IMP-positive strains displayed comparable changes to NDM strains, characterized by an increase in 1,2-ethanediol and a decrease in 3-hydroxy-2-butanone (acetoin) and 4 unidentified VOCs (Fig. 11A). Finally, a three-dimensional PCA score plot revealed a separation trend after including these VOCs, which significantly facilitated the recognition of class B CBPM-producing *K. pneumoniae* strains, especially VOCs released by NDM-positive and IMP-positive strains. (Fig. 11B).

## Discussion

The rapid and accurate identification of BSIs caused by CRKP and the characterization of CBPMs are crucial in addressing the threat posed by the rapid global epidemic of multidrug-resistant bacteria, considering the escalating detection rate of CRKP in such infections (Chang et al. 2021; Chen et al. 2022). This study has the following main findings: (1) the identification of *K. pneumoniae* can be facilitated through the relative composition of VOCs (VOC fingerprints) in BC bottles (BacT/ALERT® SA). (2) The disparity in VOCs emitted by CSKP and CRKP following the addition of IPM was further substantiated, leading to the discovery of potential indicators for the discernment of CRKP in BC bottles. (3) The inclusion of CBPM inhibitors (avibactam sodium and DPA) resulted in discernible alterations in the composition of specific VOCs in the corresponding strains, thereby offering a novel approach to the detection and characterization of CBPM phenotypes.

In this study, simulated BCs were conducted, excluding peripheral blood, due to the significant variability in blood metabolism across environmental factors and individuals (Kim et al. 2014; Nicholson et al. 2011). Meanwhile, compared with fresh medium without blood, a previous study (Rees et al. 2016) confirmed a 20% increase in the total number of *K*. *pneumoniae*-associated volatiles in blood-containing media but VOCs already produced in the media without blood were unaffected; however, these findings warrant further validation. By focusing solely on the metabolism of *K*. *pneumoniae* without the confounding influence of blood, we provide a reference point for future studies. Further, to mitigate the influence of adsorbed beads (BacT/ALERT FA Plus, Biomérieux, Nürtingen, Germany), and limited by the inability to establish an anaerobic environment in vitro, we ultimately opted for the BacT/ALERT® SA culture medium. In addition, utilizing the alteration in VOCs of the standard strains as a benchmark and those of the clinical strains as a corroborative measure undoubtedly confers an additional significant advantage to the current study.

In recent years, VOCs have been increasingly utilized for strain characterization (Drees et al. 2019; Kunze et al. 2013; Lu et al. 2022). In addition, it is possible to differentiate CRKP and CSKP in TSB media using changes in VOCs (Filipiak et al. 2022). Nevertheless, we note that there are few studies employing commercial BC bottles emitting VOCs to identify CRKP and determine its CBPM phenotype, further emphasizing the strengths of the present study.

Fifty-four VOCs were isolated in the current study, of which 18 were successfully identified using the GC–IMS technique (6 VOCs existed as both monomers and dimers). Although 30 VOCs remained unidentified, valuable information regarding these compounds was obtained (Table 1). It is anticipated that the continuous advancement of technology and further research will results in identification of additional VOCs.

Previous studies have demonstrated a correlation between the growth metabolism of *K*. *pneumoniae* and VOCs, including propionic acid (BCs) (Julak et al. 2000), acetic acid (BCs/LB) (Julak et al. 2000), 3-methyl-1-butanol (sheep blood agar/TSB/LB) (Filipiak et al. 2022; Junger et al. 2012; Luo et al. 2023), butane-2,3-dione (diacetyl) (TSB) (Filipiak et al. 2022), and 3-hydroxy-2-butanone (malt extract agar and dichloran glycerol agar/TSB supplemented with 5% hemolyzed human blood) (Kiviranta et al. 1998; Rees et al. 2016). These findings align with the outcomes of the present study. Other identified VOCs released by *K. pneumoniae*, including 3-methylbutanoic acid, 2-methylpropanoic acid, 1,2-ethanediol, isovalerone, and 2-methylpyrazine-M, yielded varying outcomes in our study than in other studies maybe due to the dissimilar nutrient composition between commercial BC bottles and other media. Notably, variations in the employed detection methods have resulted in difference in the identified VOCs in the samples. Additionally, VOCs that were readily absorbed by *K*. *pneumoniae* and exhibited high concentrations within the blank medium undergo metabolic processes, leading to their transformation into different compounds, including benzaldehyde (TSB) (Filipiak et al. 2022), butan-2-one, and 2-methylpyrazine-D. However, it is noteworthy that butane-2,3-dione (diacetyl) and butan-2-one variations in the present study differed from that in previous studies (Boots et al. 2014; Rees et al. 2017). The results demonstrated that both substances were present at high levels in the blank medium, and due to the relatively small sample size, the results of these differential studies warrant further exploration. Interestingly, consistent with a previous study (Filipiak et al. 2022), the alterations observed in the VOCs (Additional file 2: Table S2) were generally congruent with the growth trend of *K*. *pneumoniae* (Fig. 1), as evidenced by the stabilization of VOC changes after 5 h of bacterial growth (T2), further supporting our decision to prioritize the examination of VOC changes at the T2 time point.

The catabolism of leucine by *K*. *pneumoniae* through the Ehrlich pathway was shown to generate 3-methyl-1-butanol (Luo et al. 2023; Smart et al. 2019). Similarly, the observed elevation in 1,2-ethanediol levels could potentially be attributed to the metabolic processes associated with fatty acids. Meanwhile, the synthesis of 2,3-butanedione occurs through the action of bacterial decarboxylases, which convert (2*S*)-2-hydroxy-2-methyl-3-oxobutanoic acid derived from pyruvate metabolism to 2,3-butanedione (Whiteson et al. 2014). The release of 3-hydroxy-2-butanone—another significant VOC—has been linked to glycolysis process (Chen et al. 2014). In the metabolic process of pyruvate, the final product of glycolysis and the carbon source for the citric acid cycle results in the production of acetylactate, which is subsequently transformed into 3-hydroxy-2-butanone through the action of α-acetolactate decarboxylase (Chen et al. 2014). In addition, the absorption of benzaldehyde may be affected by the enzymatic reduction of benzaldehyde by benzaldehyde dehydrogenase, which catalyzes the production of nicotinamide adenine dinucleotide phosphate (NADPH) during the growth of *K*. *pneumoniae* (Filipiak et al. 2022). However, due to the lack of precise knowledge regarding the specific constituents of the media in BC bottles coupled with the intricate nature of biochemical reactions, we did not determine the possible mechanisms underlying the remaining variations in differential VOCs.

In our study, we found that CRKP and CSKP can be differentiated without inclusion of IPM, but these differences disappear over time (Additional file 3: Fig. S4B), which is consistent with previous literature (Filipiak et al. 2022; Żuchowska and Filipiak 2023). In addition, 4 VOCs could not be identified and thus their significance is unknown. The bactericidal effects of IPM have been widely documented. For instance, several studies have demonstrated that it inhibits the biosynthesis of bacterial cell walls, leading to the lysis and subsequent death of bacteria (Pai Mangalore et al. 2022). Moreover, incorporation of IPM promoted the differentiation of CSKP from CRKP, with CRKP identified based on the differential VOCs, arising from killing of the susceptible cells, as reported in a previous study (Filipiak et al. 2022). Notably, the killing of sensitive bacteria by IPM caused a significant decrease in the amount of released VOCs and an increase in the number of absorbed VOCs compared to CRKP, and this difference remained unchanged over time (Fig. 4 and Additional file 3: Fig. S6). In addition, the incorporation of IPM exhibited different metabolic profiles between the standard and clinical strains, characterized by varying VOCs compared to the CSKP, and both strains could effectively differentiate between CRKP and CSKP based on specific differential VOCs. This may be as a result of the difference in origin of the two strains, clinical strains are susceptible to antibiotic-induced mutation and have different metabolism profiles from the standard strains (Filipiak et al. 2022). However, this study and our previous one (Luo et al. 2023) have consistently demonstrated the challenging nature of identifying carbapenemase-negative CRKP, which calls for further refinement of our experimental protocols.

The presence of VOCs in CRKP strains following the addition of IPM (4 h) has been documented (Filipiak et al. 2022). However, we did not obtain similar results, probably due to the limited duration of IPM addition and limitations of detection methodology, it provides valuable insights. Our findings suggest that alterations in VOCs may serve as a potential means to identify CRKP, opening new avenues for diagnosis and treatment options. Initially, we found that the inclusion of the enzyme inhibitor 3-aminophenylboronic acid (dissolved in dimethyl sulfoxide) increased the difficulty of identifying VOCs. Moreover, the alkaline nature (pH = 8.0) of EDTA also interfered with the outcomes. Consequently, avibactam sodium and DPA were selected as alternative components in the subsequent experiments. It has been demonstrated that avibactam is an effective inhibitor of class A, class C, and certain class D enzymes (Coleman 2011). In our study, the combination of averbactam sodium and IPM effectively eradicated *K. pneumoniae* strains that generate class A enzymes (standard and clinical strains). Furthermore, this treatment altered the levels of VOCs in class A enzyme-producing *K. pneumoniae* strains, which provided a reliable method for detecting it. However, our findings demonstrate that the addition of avibactam sodium was not effective in treating *K. pneumoniae* strains producing class D enzymes, which may be attributed to the inadequate avibactam sodium concentration. Pyridine-2,6-dicarboxylic acid (DPA), as an important type of Metallo-β-lactamases (MBL) inhibitor, can inhibit MBL by chelating, stripping, and binding Zn^2+^ in the active center of MBL (Chen et al. 2017a; Wang et al. 2020). Similar to the effects of avibactam sodium, DPA showed the capacity to selectively alter the VOCs of *K. pneumoniae* strains which produce class B enzymes, providing a novel approach for the identification of class B enzymes.

Although the findings presented in this preliminary study are interesting, several limitations should be acknowledged. The study mainly relied on clinical strains obtained from a single hospital which restricts the generalizability of the results to some extent. This limitation needs to be considered when interpreting the findings and their implications. In the future, we aim to expand the samples to detect more carbapenemase types of CRKP. Secondly, the experiments conducted to simulate blood cultures did not account for the impact of blood samples on the detection of VOCs. Studies have shown that incorporation of blood in these experiments increases the production of VOCs (Rees et al. 2016), and this needs to be further investigated. Thirdly, the use of single detection technique also limited the significance of our results. Although we successfully detected 54 VOCs, the identification was limited to 18 VOCs, of which 6 VOCs existed as both monomers and dimers. Therefore, it is imperative to incorporate additional detection methods to comprehensively analyze the fluctuations in VOCs. Lastly, the experimental designs and methods need to be further revised and improved. This will be the focus of our future investigations.

In conclusion, using GC–IMS technology, we identified CRKP strains by analyzing changes in VOC. In addition, we investigated the potential application of VOCs in the detection of phenotypes within CRKP strains. Moreover, we developed VOC fingerprints to facilitate the identification of relevant strains. Nevertheless, we acknowledge that these findings need to be further validated through large-scale, multi-center, prospective experiments.

### Supplementary Information

**Additional file 1: Table S1.** The experimental parameters of GC–IMS.

**Additional file 2: Table S2.** Temporal changes in VOCs measured by GC–IMS (without imipenem added, standard strains). **Table S3.** The change of VOCS in T2 time period compared with the blank control group (without imipenem added, standard strains, mean ± SD). **Table S4.** Temporal changes in VOCs measured by GC–IMS (with imipenem added, standard strains). **Table S5.** The change of VOCs in T2 time point compared with the CSKP group (with imipenem added, standard strains, mean ± SD). **Table S6.** The change of VOCs in T2 time point after adding enzyme inhibitors treatment (realtive volume of VOC, mean ± SD, standard strains). **Table S7.** The change of VOCS in T2 time point compared with the CSKP group (with imipenem added, clinical isolates, mean ± SD). **Table S8.** The change of VOCs in T2 time point after adding enzyme inhibitors treatment (realtive volume of VOC, mean ± SD, clinical isolates).

**Additional file 3: Figure S1.** A schematic diagram of GC–IMS. **Figure S2.** The flow chart of the sample preparation. Some images (blood agar plate and liquid transfer gun) in Fig. S2 were free and adapted from Servier Medical ART (https://smart.servier.com). **Figure S3.** The flow chart of the workflow of GC–IMS. **Figure S4.** The differential VOCs (CSKP vs. CRKP) produced by *K. pneumoniae* (standard strains, without IPM added). **A** Comparison of the differential VOCs in the indicated groups. **B** The temporal changes in CRKP-characterized VOCs (T0–T4). **Figure S5.** The correlation heatmap of differential VOCs in the CRKP group (at T2, standard strains, with IPM added). **Figure S6.** The heatmap of the temporal changes in CRKP-characterized VOCs (T0-T4, after min–max normalization). **A** ATCC BAA-1706. **B** ATCC BAA-1705. **C** ATCC BAA-2146. **D** ATCC BAA-2524. **Figure S7.** The heatmap demonstrating the results of experiments repeated six times, illustrating consistent and replicable outcomes (after min–max normalization). **Figure S8.** The growth curve of *K. pneumoniae* (clinical strains). **Figure S9.** The heatmap of the differentially expressed VOCs constructed using the volcano plots (compared with the CSKP group, after binarization).

## Data Availability

The datasets generated during and/or analysed during the current study are available from the corresponding author on reasonable request.

## Abbreviations

GC–IMS: Gas chromatography–ion mobility spectrometry headspace
CRKP: Carbapenem-resistant *Klebsiella pneumoniae*
VOCs: Volatile organic compounds
BCs: Blood cultures
*K. pneumoniae*: *Klebsiella pneumoniae*
CSKP: Carbapenem-susceptible *Klebsiella pneumoniae*
BSI: Bloodstream infection
DIC: Disseminated intravascular coagulation
CRE: Carbapenem-resistant Enterobacteriaceae
BRICS: Blood Bacterial Resistance Investigation Collaborative System
CIM: Carbapenem inactivation method
CBPM: Carbapenemase
TSB: Trypticase soy broth
GC–MS: Gas chromatography–mass spectrometry
GEP: Gas electrophoresis
PEC: Plasma chromatography
ATCC: American Type Culture Collection
MALDI-TOF/MS: Matrix-assisted laser desorption/ionization time-of-flight mass spectrometry
KB: Kirby–Bauer
ERT: Ertapenem
CLSI: The Clinical Laboratory Standards Institute
mCIM: The modified carbapenem inactivation method
eCIM: The ethylenediaminetetraacetic acid (EDTA)-modified carbapenem inactivation method
PCR: Polymerase chain reaction
CFU: Colony forming units
DPA: Pyridine-2,6-dicarboxylic acid
RI: Retention index
RIP: Reactant ion peak
FC: Fold change
