# Supplementary material for: GC–IMS facilitates identification of carbapenem-resistant Klebsiella pneumoniae in simulated blood cultures
Source: AMB Express. 2024 Apr 24;14:40. doi: 10.1186/s13568-024-01708-1 (PMC11043319; doi:10.1186/s13568-024-01708-1)
Supplement: Supplementary file 1 — Additional file 1: Table S1. The experimental parameters of GC–IMS. [file 13568_2024_1708_MOESM1_ESM.docx]

**Table S1** The experimental parameters of GC-IMS.

| The automatic headspace sampler (G.A.S, Dortmund, Germany) | |
| --- | --- |
| Volume of sample (culture medium) | 500 µL |
| Incubation time | 3 min |
| Incubation temperature | 60 ^o^C |
| Rotating speed for incubating | 500 rpm |
| Injector temperature | 85 ^o^C |
| GC-IMS (FlavourSpec®; G.A.S., Dortmund, Germany) | |
| Volume of headspace gas | 1 mL |
| Temperature of drift tube (T1) | 45 ^o^C |
| Temperature of chromatographic column (T2) | 80 ^o^C |
| Temperature of injection port (T3) | 80 ^o^C |
| Temperature of joints (T4, T5) | T4: 80 ^o^C; T5:45 ^o^C |
| Chromatographic column | MXT-WAX column (15 m x 0.53 mm x 0.1 μm) |
| Carrier gas and drift gas | Nitrogen of 99.99% purity |
| Ionization source | Tritium source (3H) |
| Average radiation energy | 5.68 keV |
| Drift tube length | 98 mm |
| Ionization mode | Positive ionization mode |
| Flow of drift tube (EPC1) | 150 mL/min |
| Flow of chromatographic column (EPC2) | For details see the general remarks |
| Total analysis time | 10 min |

Note: GC-IMS with increasing analysis time, flow ramps were used (EPC2). Initially, the carrier gas (nitrogen, 99.99% purity) flow rate was 2 mL/min (0-3 min). Then, the carrier gas flow rate was 10 mL/min (3-10 min). Finally, the carrier gas flow rate was 100 mL/min (when the analysis of GC-IMS finished).
