# Supplementary material for: GC–IMS facilitates identification of carbapenem-resistant Klebsiella pneumoniae in simulated blood cultures
Source: AMB Express. 2024 Apr 24;14:40. doi: 10.1186/s13568-024-01708-1 (PMC11043319; doi:10.1186/s13568-024-01708-1)
Supplement: Supplementary file 3 — Additional file 3: Figure S1. A schematic diagram of GC–IMS. Figure S2. The flow chart of the sample preparation. Some images (blood agar plate and liquid transfer gun) in Fig. S2 were free and adapted from Servier Medical ART (https://smart.servier.com). Figure S3. The flow chart of the workflow of GC–IMS. Figure S4. The differential VOCs (CSKP vs. CRKP) produced by K. pneumoniae (standard strains, without IPM added). A Comparison of the differential VOCs in the indicated groups. B The temporal changes in CRKP-characterized VOCs (T0–T4). Figure S5. The correlation heatmap of differential VOCs in the CRKP group (at T2, standard strains, with IPM added). Figure S6. The heatmap of the temporal changes in CRKP-characterized VOCs (T0-T4, after min–max normalization). A ATCC BAA-1706. B ATCC BAA-1705. C ATCC BAA-2146. D ATCC BAA-2524. Figure S7. The heatmap demonstrating the results of experiments repeated six times, illustrating consistent and replicable outcomes (after min–max normalization). Figure S8. The growth curve of K. pneumoniae (clinical strains). Figure S9. The heatmap of the differentially expressed VOCs constructed using the volcano plots (compared with the CSKP group, after binarization). [file 13568_2024_1708_MOESM3_ESM.pdf]

Additional file 3

Figure S1. A schematic diagram of GC–IMS.

Figure S2. The flow chart of the sample preparation. Some images (blood agar plate and liquid transfer gun) in Fig. S2 were free and adapted from Servier Medical ART (https://smart.servier.com).

Figure S3. The flow chart of the workflow of GC–IMS.

Figure S4. The differential VOCs (CSKP vs. CRKP) produced by *K. pneumoniae* (standard strains, without IPM added). **A** Comparison of the differential VOCs in the indicated groups. **B** The temporal changes in CRKP-characterized VOCs (T0–T4).

Figure S5. The correlation heatmap of differential VOCs in the CRKP group (at T2, standard strains, with IPM added).

Figure S6. The heatmap of the temporal changes in CRKP-characterized VOCs (T0-T4, after min–max normalization). **A** ATCC BAA-1706. **B** ATCC BAA-1705. **C** ATCC BAA-2146. **D** ATCC BAA-2524.

Figure S7. The heatmap demonstrating the results of experiments repeated six times, illustrating consistent and replicable outcomes (after min–max normalization).

Figure S8. The growth curve of *K. pneumoniae* (clinical strains).

Figure S9. The heatmap of the differentially expressed VOCs constructed using the volcano plots (compared with the CSKP group, after binarization).

Figure S1

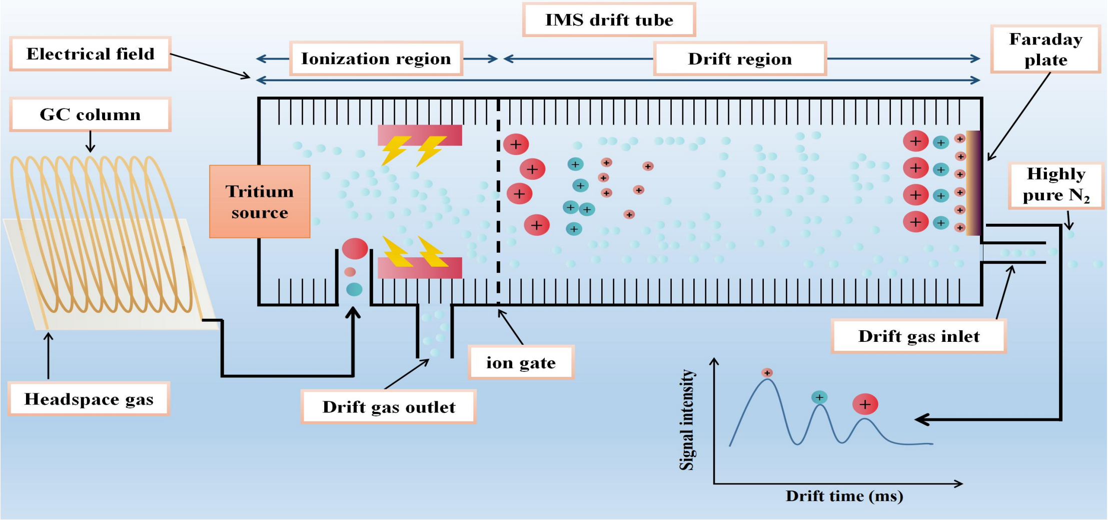

Figure S2

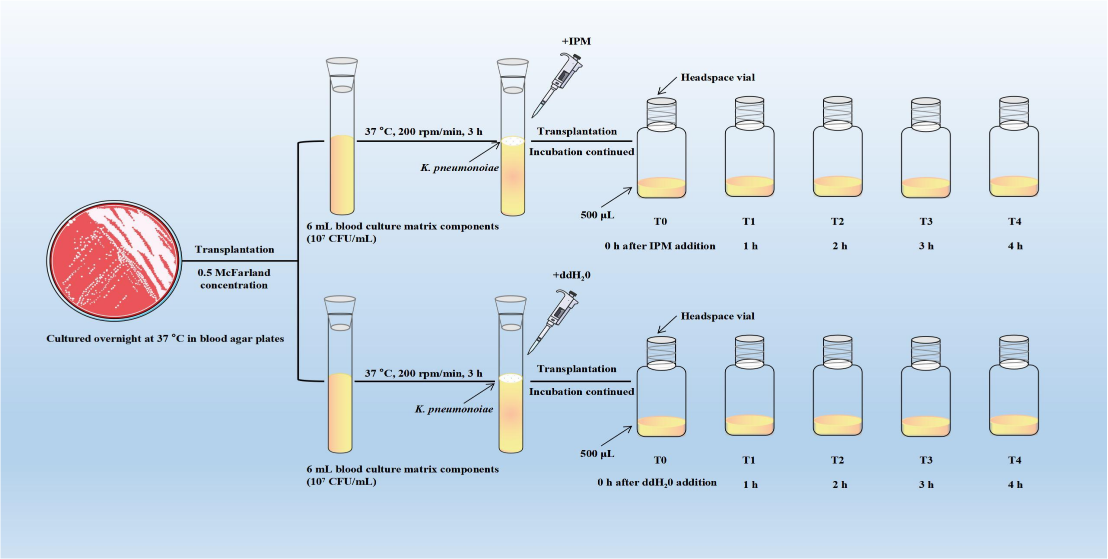

Figure S3

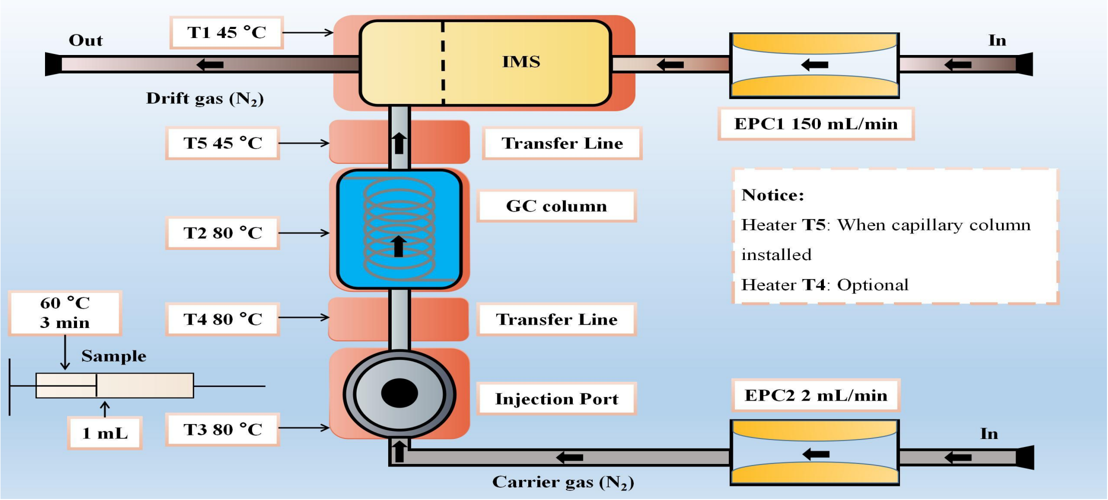

Figure S4

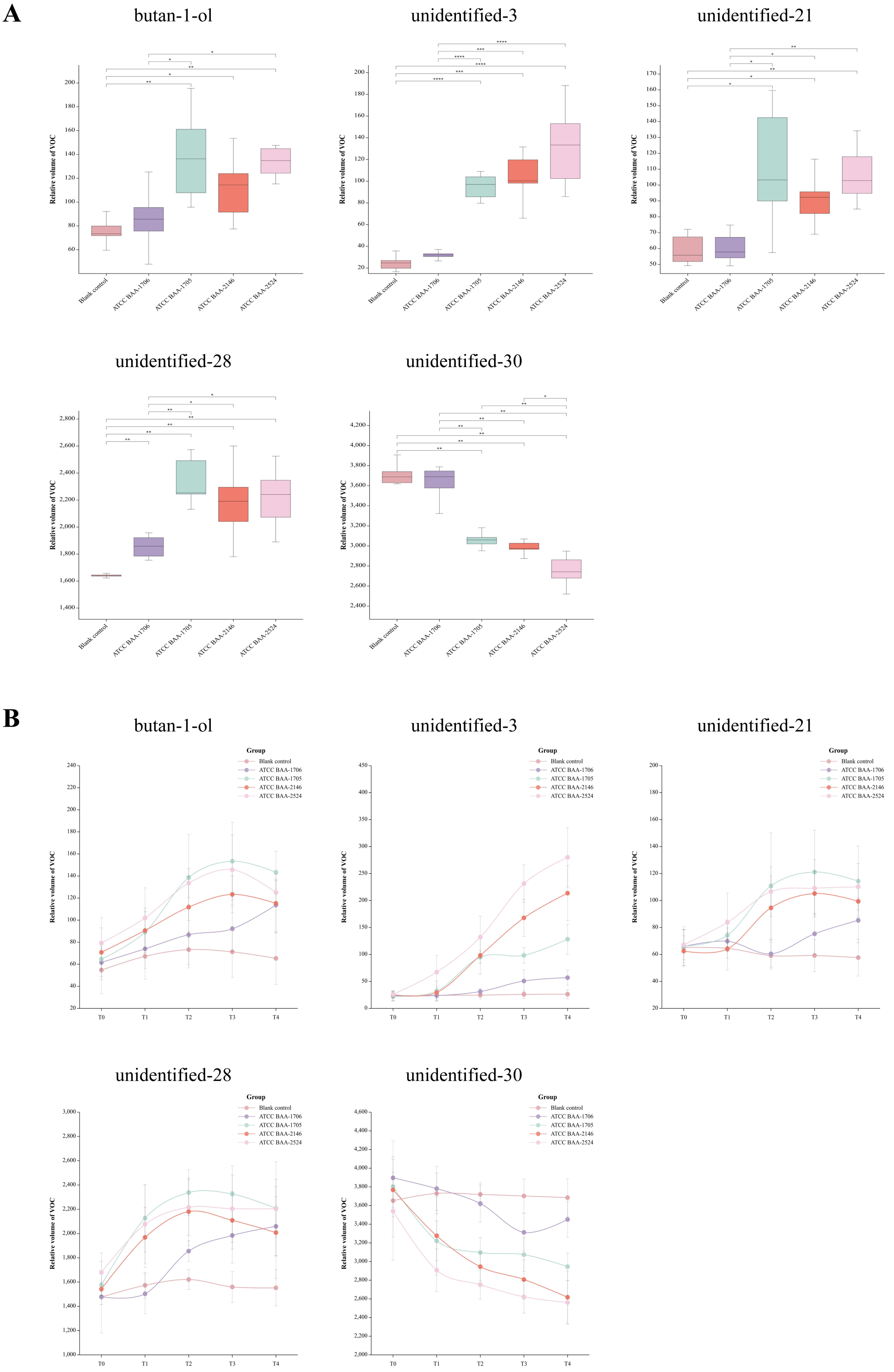
A
butan-1-ol
unidentified-3
unidentified-21
unidentified-28
unidentified-30
Relative volume of VOC
Blank control
ATCC BAA-1706
ATCC BAA-1705
ATCC BAA-2146
ATCC BAA-2524
B
butan-1-ol
unidentified-3
unidentified-21
unidentified-28
unidentified-30
Group
Blank control
ATCC BAA-1706
ATCC BAA-1705
ATCC BAA-2146
ATCC BAA-2524
Relative volume of VOC
T0
T1
T2
T3
T4

Figure S5

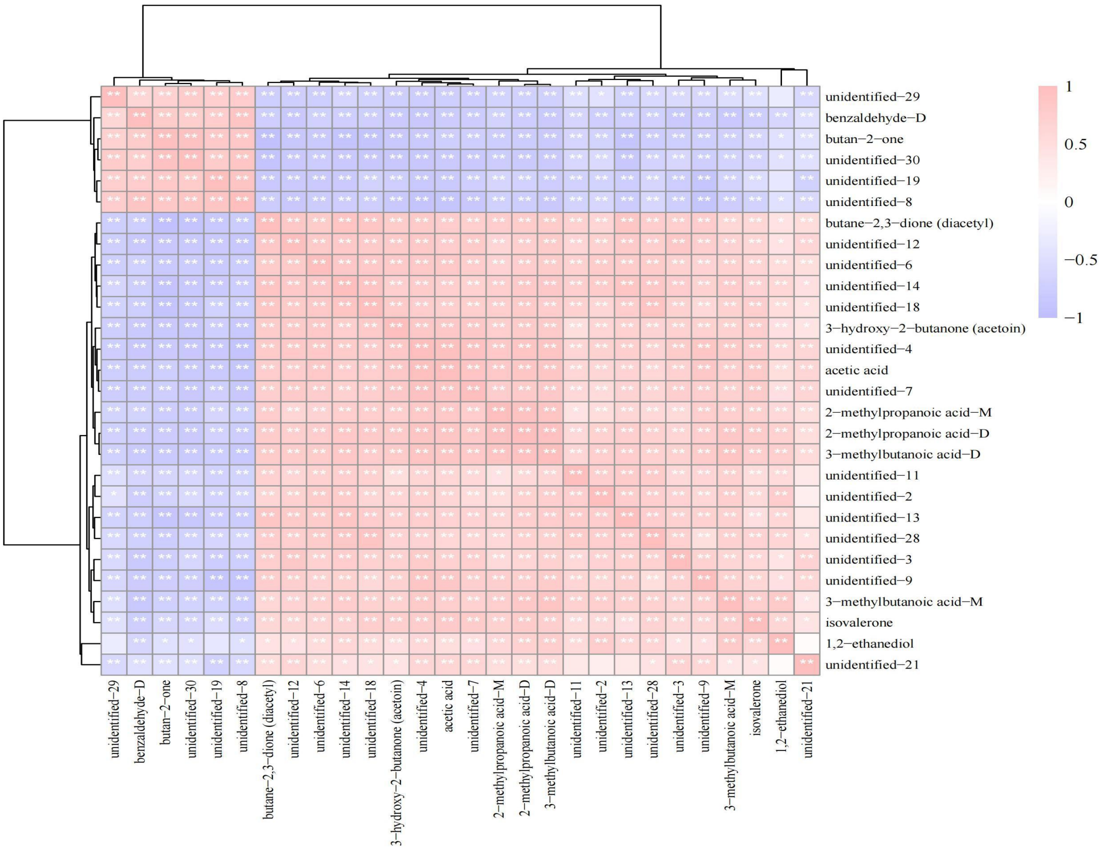

Figure S6

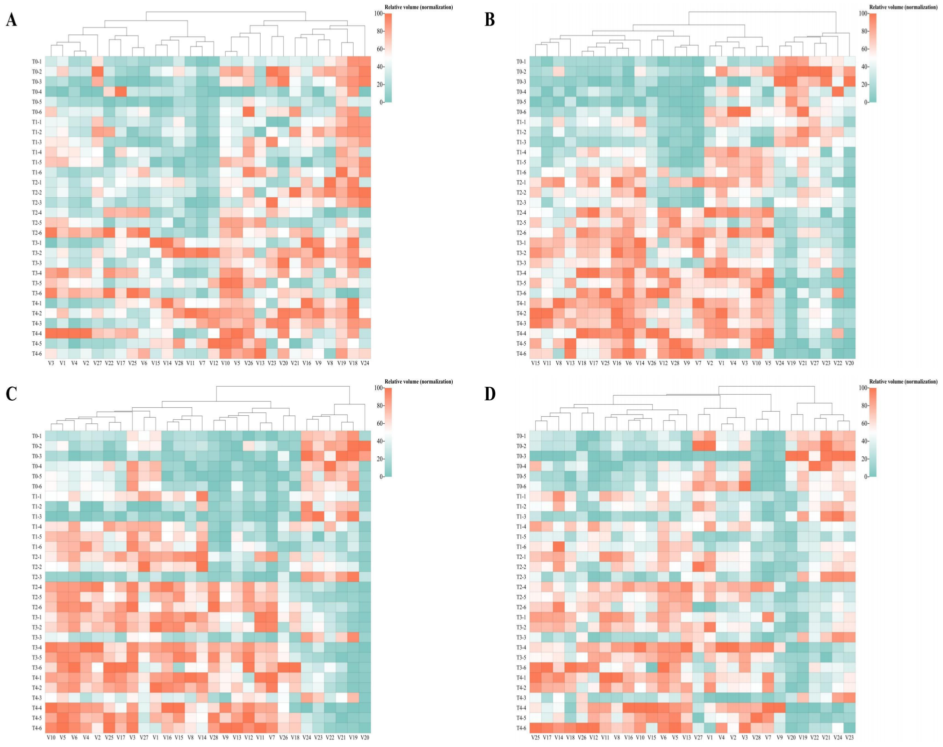

Label	VOC
V1	3-methylbutanoic acid-M
V2	3-methylbutanoic acid-D
V3	2-methylpropanoic acid-M
V4	2-methylpropanoic acid-D
V5	acetic acid
V6	butane-2,3-dione (diacetyl)
V7	3-hydroxy-2-butanone (acetoin)
V8	unidentified-2
V9	unidentified-3
V10	unidentified-4
V11	unidentified-6
V12	unidentified-7
V13	unidentified-9
V14	unidentified-11
V15	unidentified-13
V16	unidentified-14
V17	unidentified-18
V18	unidentified-28
V19	butan-2-one
V20	benzaldehyde-D
V21	unidentified-8
V22	unidentified-19
V23	unidentified-29
V24	unidentified-30
V25	isovalerone
V26	unidentified-21
V27	1,2-ethanediol
V28	unidentified-12

Figure S7

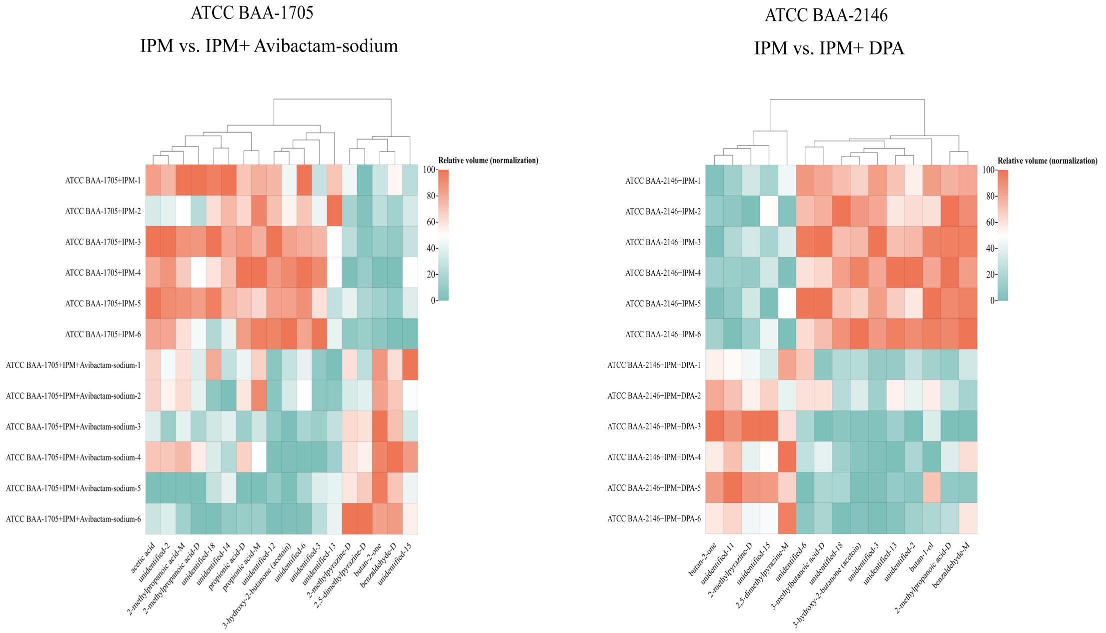

Figure S8

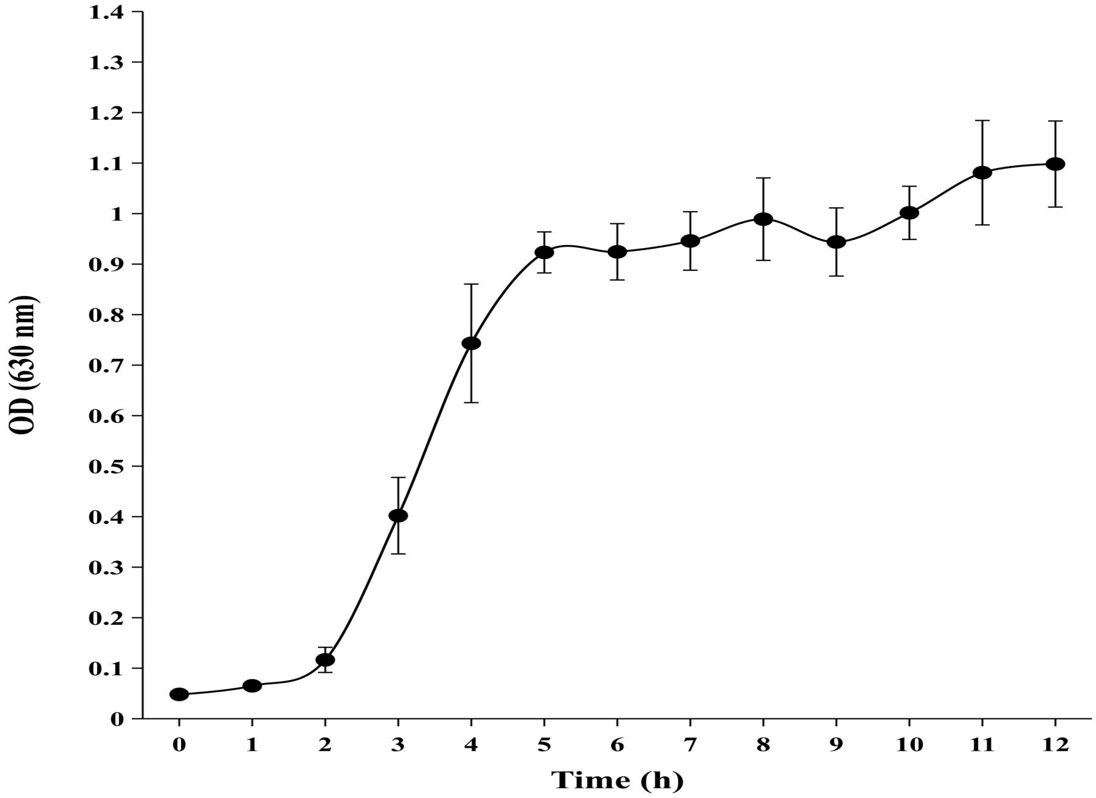

Figure S9

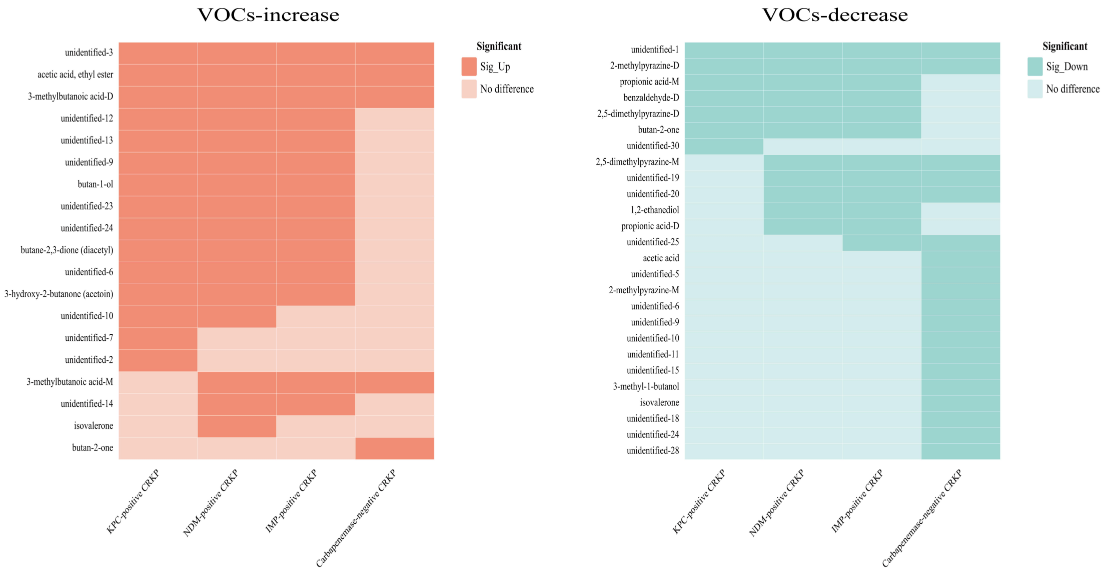
VOCs-increase
Significant
Sig_Up
No difference
unidentified-3
acetic acid, ethyl ester
3-methylbutanoic acid-D
unidentified-12
unidentified-13
unidentified-9
butan-1-ol
unidentified-23
unidentified-24
butane-2,3-dione (diacetyl)
unidentified-6
3-hydroxy-2-butanone (acetoin)
unidentified-10
unidentified-7
unidentified-2
3-methylbutanoic acid-M
unidentified-14
isovalerone
butan-2-one
KPC-positive CRKP
NDM-positive CRKP
IMP-positive CRKP
Carbapenemase-negative CRKP
VOCs-decrease
Significant
Sig_Down
No difference
unidentified-1
2-methylpyrazine-D
propionic acid-M
benzaldehyde-D
2,5-dimethylpyrazine-D
butan-2-one
unidentified-30
2,5-dimethylpyrazine-M
unidentified-19
unidentified-20
1,2-ethanediol
propionic acid-D
unidentified-25
acetic acid
unidentified-5
2-methylpyrazine-M
unidentified-6
unidentified-9
unidentified-10
unidentified-11
unidentified-15
3-methyl-1-butanol
isovalerone
unidentified-18
unidentified-24
unidentified-28
KPC-positive CRKP
NDM-positive CRKP
IMP-positive CRKP
Carbapenemase-negative CRKP
